# Effects of maternal calcium supplementation on offspring blood pressure and growth in childhood and adolescence in a population with a low-calcium intake: follow-up study of a randomized controlled trial

**DOI:** 10.1016/j.ajcnut.2024.02.025

**Published:** 2024-04-18

**Authors:** Ann Prentice, Landing MA Jarjou, Gail R Goldberg, Simon M Schoenbuchner, Sophie E Moore, Kate A Ward, Tim J Cole

**Affiliations:** 1 MRC Unit The Gambia at London School of Hygiene and Tropical Medicine, Serrekunda, The Gambia; 2 MRC Nutrition and Bone Health Research Group, Cambridge, United Kingdom; 3 MRC Epidemiology Unit, University of Cambridge, Cambridge, United Kingdom; 4 Centre for Trials Research, Cardiff University, Cardiff, United Kingdom; 5 MRC Lifecourse Epidemiology Centre, University of Southampton, Southampton, United Kingdom; 6 Department of Women and Children’s Health, Kings College London, London, United Kingdom; 7 Department of Population, Policy and Practice Research and Teaching, University College London Great Ormond Street Institute of Child Health, London, United Kingdom

**Keywords:** adolescence, Africa, anthropometry, blood pressure, calcium, dietary supplements, Gambia, growth, pregnancy, pubertal maturation

## Abstract

**Background:**

The World Health Organization recommends calcium supplementation (1500–2000 mg/d) during pregnancy for women with a low-calcium intake.

**Objectives:**

The purpose of this study was to investigate whether pregnancy calcium supplementation affects offspring blood pressure and growth in The Gambia where calcium intakes are low (300–400 mg/d).

**Methods:**

Follow-up of offspring born during a randomized controlled trial of pregnancy calcium supplementation (ISRCTN96502494, 1996–2000) in which mothers were randomly assigned to 1500 mg Ca/d (Ca) or placebo (P) from 20 wk pregnancy to delivery. Offspring were enrolled at age 3 y in studies where blood pressure and anthropometry were measured under standardized conditions at approximately 2-yearly intervals. Mean blood pressure and growth curves were fitted for females and males separately, using the longitudinal SuperImposition by Translation and Rotation (SITAR) mixed effects model. This generates 3 individual-specific random effects: size, timing, and intensity, reflecting differences in size, age at peak velocity, and peak velocity through puberty relative to the mean curve, respectively.

**Results:**

Five hundred twenty-three singleton infants were born during the trial (maternal group assignment: Ca/P = 259/264). Four hundred ninety-one were enrolled as children (females: F-Ca/F-P = 122/129 and males: M-Ca/M-P = 119/121) and measured regularly from 3.0 y to mean age 18.4 y; 90% were measured on ≥8 occasions. SITAR revealed differences in the systolic blood pressure and height curves between pregnancy supplement groups in females, but not in males. F-Ca had lower systolic blood pressure than F-P at all ages (size = −2.1 ± SE 0.8 mmHg; *P* = 0.005) and lower peak height velocity (intensity = −2.9 ± SE 1.1%, *P* = 0.009). No significant pregnancy supplement effects were seen for other measures.

**Conclusions:**

This study showed, in female offspring, that pregnancy calcium supplementation may lower systolic blood pressure and slow linear growth in childhood and adolescence, adding to evidence of offspring sexual dimorphism in responses to maternal supplementation. Further research is warranted on the long-term and intergenerational effects of antenatal supplementations.

This trial was registered at ISRCTN Registry as ISRCTN96502494.

## Introduction

WHO recommends calcium supplementation at 1500–2000 mg elemental calcium/day (mg Ca/d) for pregnant women in populations with a low-calcium intake to reduce the risk of pre-eclampsia and its complications [1]. These recommendations were based on a series of randomized controlled trials (RCTs) with maternal pregnancy outcomes as endpoints. In RCTs conducted in the United States and Argentina, pregnancy calcium supplementation with 2000 mg Ca/d was further shown to reduce the systolic blood pressure (SBP) of the offspring at ages 2 y and 7 y, respectively [2,3], suggesting that there may be long-lasting effects in the offspring of calcium supplements taken by the mother during pregnancy.

Blood pressure tracks from childhood through to adult life [4]. In low- and middle-income countries, there has been an increase in population mean blood pressure and the prevalence of hypertension, particularly in Sub-Saharan Africa [5,6]. Hypertension is one of the leading risk factors for death and disability in the older population globally [7], and stroke, the major clinical outcome of uncontrolled hypertension, is a leading risk of death in Africa [8]. The risk factors associated with elevated blood pressure in childhood include prenatal nutrient restriction, fetal growth restriction leading to low-birth weight, and accelerated growth in the first years of life [[9], [10], [11], [12]], often common experiences in low- and middle-income countries. The tracking of blood pressure from childhood suggests that pregnancy calcium supplementation may provide a window of opportunity for intervention to reduce offspring hypertension in adulthood in populations with a low-customary calcium intake.

The Gambia, West Africa, has high rates of adult hypertension and stroke, in both urban and rural communities [13,14]. Prenatal nutrient restriction and accelerated growth after periods of growth faltering in early life are common [15,16]. In an RCT in rural Gambia, a population with a low-calcium intake (300–400 mg/d), a maternal supplement of 1500 mg Ca/d from 20 wk gestation to term had no significant effect on maternal blood pressure, birth outcomes, or infant growth [17,18] and no apparent effect on offspring blood pressure at 7 y of age [19]. However, the maternal supplement had altered the growth and bone development of the offspring, as evidenced at age 8–12 y [20], and in ways that differed between females and males. Female offspring of mothers in the calcium group had slower growth compared with the placebo group, whereas male offspring of calcium-supplemented mothers had accelerated growth [20]. This suggests that there may be unexpected effects of pregnancy calcium supplementation on the offspring that differ between females and males. The aim of the study presented here was to investigate this possibility further using a collection of repeated offspring blood pressure and anthropometric measurements from 3 y to young adulthood.

## Methods

### Participants and study design

The participants were children born to mothers who had participated in an RCT of calcium supplementation during pregnancy (ISCRTN96502494) and who had their blood pressure and anthropometry measured repeatedly from the age of 3 y during childhood and adolescence in a series of connected but separately designed and approved follow-up studies conducted from 1998–2018. Scientific and ethics approvals for these studies were granted by the Gambia Government/Medical Research Council (MRC) Joint Ethics Committee; full details are provided in Supplemental Table 1. Fully informed written consent for each of the studies was obtained from the parents or guardians of the children after a verbal explanation was given in the parents’ language. Once the offspring were aged 18 y, they provided their own written consent. All measurements were made with the assent of the child. Throughout the studies, the investigators and research staff in The Gambia and United Kingdom remained blinded to the maternal supplementation group allocation except the code holder (AP) who was not involved in data collection.

The design of the pregnancy calcium supplementation trial and the primary outcomes for the mothers and infants have been reported elsewhere [17,18,21]. In brief, pregnant mothers who presented at antenatal clinics run by the MRC in 16 rural villages in West Kiang, The Gambia, between May 1995 and February 2000 were invited to participate in an RCT of supplementation with 1500 mg Ca/d as calcium carbonate (3 orange-flavored chewable tablets Calcichew, Nycomed Pharma AS; distributed in the United Kingdom by Shire Pharmaceutical Development Ltd.) or cellulose–lactose placebo (Nycomed Pharma AS) from 20 wk of pregnancy until delivery. Pregnant mothers were randomly assigned at enrollment, stratified according to the 3 clusters of villages served by different midwives. Randomization was by permuted blocks of 4 to ensure an even distribution of supplement groups across the different seasons and years. Compliance with the trial, measured by tablet count, was 97% and did not differ between the groups. The mean calcium intake prior to supplementation measured in a subset of mothers was 345 ± SD 206 mg/d in the calcium-supplemented group and 363 ± SD 174 mg/d in the placebo group [17,18].

Of the 662 mothers enrolled, 523 completed the trial and delivered a healthy, singleton infant (264 females and 259 males) that survived the neonatal period. Included in this total are 3 infants of mothers whose blood pressure was not measured at 36 weeks of pregnancy and therefore not included in the published account of the trial’s primary outcomes [18]. Excluded are 5 infants of mothers who were shown subsequently to have been enrolled later than 24 wk pregnancy. The proportion of female and male babies born did not differ by maternal supplement group, season, or midwife cluster [18].

These 523 children formed the base set eligible to participate in the follow-up studies. They were traced before they reached their third birthday and invited to enroll in the Pregnancy Study Offspring cohort (PSO). Of these, 26 had died, 6 were either ill or away from The Gambia, and 491 were enrolled. The studies in which blood pressure measurements and anthropometry were performed on members of this cohort are described below. Participants who were unavailable for any of the measurement rounds, usually because of schooling or illness, were included among those invited to attend subsequently, unless they had withdrawn from future participation, left The Gambia, or died.

The children in the cohort were invited to participate in a total of 13 regular follow-up sessions until their late teenage years. These were part of 3 separately approved studies with different primary outcomes where blood pressure and anthropometric data were also collected (Supplemental Table 1). The ages of the children, calendar years when measurements were made, and numbers measured at each of the sessions are shown graphically in Supplemental Table 2. The studies were as follows:

#### PSO

The primary outcomes for this longitudinal study were blood pressure and anthropometry measured on or near the child’s third birthday (PSO.Y3) and every 2 y subsequently (PSO.Y5 to PSO.Y17), covering the period October 1998 to March 2016. All measurements were performed at a convenient location in the family home, compound, or local clinic by trained members of the research team. Children who had moved from their original village but remained in The Gambia were located and measured in their new home.

#### PSE.B

This cross-sectional study, known as Pregnancy Study Early Nutrition Project; Cohort B and conducted between November 2005 and August 2006, was a component of a multicohort project designed to investigate the association between early-life nutrition and cardiovascular disease risk and included blood pressure measurements and anthropometry [19,22,23]. Members of the PSO cohort who were still residing in West Kiang or had moved but were living within 1 h of the MRC Laboratories in the coastal urban area (*n* = 452) were eligible to participate; 389 were enrolled and measured at age 5–10 y [19]. The measurements were conducted in the family home or compound.

#### PSC.B, PSC.F1, PSC.F2, and PSC.F3

The primary objective of this longitudinal series, known as Pregnancy Study Children's Bone Health (PSC) Baseline and Follow-Up Studies and conducted between May 2007 and August 2018, was to chart the development of the skeleton during adolescence, using dual-energy X-ray absorptiometry, peripheral quantitative computed tomography, and anthropometry [20]. Blood pressure measurements were included in the follow-up studies. Members of the PSO cohort were invited to participate; 447 were enrolled and measured in the baseline study (PSC.B) at age 7.8–11.9 y [20]. Fourteen additional participants were enrolled in the follow-up series (PSC.F), which were conducted ∼5 y after PSC.B (PSC.F1) and at 2-yearly intervals thereafter (PSC.F2; PSC.F3), with the oldest participant measured aged 22.4 y. Assessments were conducted at the MRC bone imaging facilities in either Keneba or Fajara for participants residing in the rural and coastal urban areas, respectively. Where there was close overlap in timing between PSC.F and PSO sessions for an individual, attendance at PSC.F was prioritized, resulting in fewer numbers recorded as having attended PSO sessions, especially at PSO.Y13 and PSO.Y17 (Supplemental Table 2).

### Blood pressure measurements

SBP and diastolic blood pressure (DBP) were measured using automated instruments: a Dinamap 8100 (Critikon Ltd.) in PSO and an Omron 7051T (Morton Medical Ltd.) in PSE.B and the PSC series. The protocol for the blood pressure measurements was highly standardized, in accordance with the manufacturers’ instructions and international best practice [24]. Measurements were carried out in a quiet environment away from distractions, generally early in the day before breakfast, using a standardized protocol. The children were put at ease by the research team, who were mostly from the same community, and measurements were performed in the presence of an adult family member. Participants were seated with legs uncrossed and both feet flat on the ground or on a footrest. Younger participants were seated on the lap of the mother or adult family member. A cuff of the appropriate size was placed on the right arm at the level of the heart with the elbow bent, upper arm straight, and lower arm at a right angle with the palm facing upward. The child then rested comfortably for 5 min. At each visit, the procedure was explained so that there was no alarm when the cuff started to inflate. Three measurements were made at 2-min intervals. A repeat set of measurements was made if the difference in SBP between the measurements was >5 mmHg. Any participant with unusually high readings was referred to a physician at the MRC clinics. For the analysis presented here, the mean of the second and third readings was used, in line with measurements made in the original pregnancy supplement (PS) trial [18]. However, the results were not materially different if the mean or the median of all 3 measurements were used.

### Anthropometry

Weight with light clothing and no shoes was measured on a flat surface using an electronic scale (Tanita HD310) to the nearest 0.1 kg. Standing height was measured with bare feet and head to the nearest 0.1 cm using a stadiometer (SECA 225) with the participant’s back to the graduated slide and chin slightly raised. BMI was calculated as weight divided by the square of height (kg/m^2^). Weight-for-age and height-for-age SD-scores were calculated using UK 1990 reference data [25]. Mid-upper arm circumference (MUAC) and triceps skinfold thickness (TST) were both measured at the upper left arm, using a nonstretchable tape measure and Holtain calipers (Holtain Ltd.). Head circumference (HC) was also measured using the nonstretchable tape. Early-life weights and lengths measured during the original trial were obtained from the trial database.

### Pubertal status assessment

Pubertal status was recorded during sessions of the PSC series. Participants were asked to compare themselves to drawings depicting the 5 Tanner Stages for breast/genital size and pubic hair development and report the nearest match [26,27]. Age of menarche was recorded by asking the participant or her mother whether menstruation had commenced and, if so, to recall how long ago and at what age the first menstrual period occurred.

### Dietary assessment

The dietary intake of participants in the PSC series was assessed by weighed intake over 2 d.

This was conducted by research staff who visited the individual at home regularly during the period of assessment to weigh foods before and after meals. Particular attention was paid to recording snacks, beverages, and calcium-rich ingredients, as described elsewhere [17,18]. An in-house Gambian food composition database linked to a version of the computer program ‘Diet In Nutrients Out’ (DINO) was used to compute daily energy and nutrient intakes. An open access version of Gambian DINO is available [28].

### Statistical analysis

To test the hypothesis of different effects of pregnancy calcium supplementation on female and male offspring, statistical analysis was conducted with the sexes separate. This resulted in 4 sex-PS groups: females of mothers in the calcium group (F-Ca), females of mothers in the placebo group (F-P), males of mothers in the calcium group (M-Ca), and males of mothers in the placebo group (M-P). The sexes were combined to test for sex differences and sex by PS interactions.

Data cleaning and initial exploratory statistical analyses were performed using DataDesk 6.3 software (Data Description Inc). Descriptive statistics at each timepoint are provided as mean ± SD, differences between groups as mean ± SE. Cross-sectional analyses at each timepoint were conducted to investigate potential effects of PS group on blood pressure and growth in each sex, using analysis of covariance with age adjustment. Relationships with current height, weight, and BMI were explored at each timepoint using linear regression.

To test the primary hypotheses of this study using longitudinal analysis, the serial data in individuals were plotted against age and summarized as smooth curves using the SuperImposition by Translation and Rotation (SITAR) mixed effects growth curve model [29] in R version 4.3.0 software. In this way, differences in curve shape between the Ca and P groups could be tested. SITAR is a shape-invariant model which estimates the mean curve as a natural cubic spline and tailors it to individuals by fitting 3 subject-specific random effects called size, timing, and intensity. Because it estimates a single curve, SITAR is robust to missing data and drop-out because where data are missing, the model borrows strength from other measurements, i.e., from the same and other individuals. The random effects shift and scale the mean curve so as to best fit the individual curves. The random effects are interpreted as follows: *size* is a random intercept, shifting the mean curve up/down to reflect the individual’s mean size; *timing* reflects the individual’s age at peak velocity during the pubertal growth spurt, shifting the mean curve left or right; and *intensity* is a multiplier that shrinks or stretches the age scale making the curve steeper or shallower; a positive (negative) intensity indicates a steeper (shallower) curve, expressed as the percentage change in the individual’s peak velocity during puberty.

In addition, the models included fixed effects that applied to all individuals, in particular an indicator variable contrasting the Ca group with the P group. Fixed effects were also included in the blood pressure models to compare the Omron to the Dinamap instrument. The Omron instrument gave appreciably lower readings than the Dinamap, by 1.0 ± SE 0.3 mmHg for SBP and 2.4 ± SE 0.3 mmHg for DBP. This was adjusted for in the models and is not discussed further.

The models were fitted assuming an unstructured correlation matrix, and data were assumed to be missing at random. The fit of each model to the data was optimized by selecting the spline curve degrees of freedom to minimize the Bayesian information criterion. The fixed effects estimated mean differences (with SEs) in size, timing, and intensity between the PS groups for each sex. Models that failed to converge were simplified by omitting the timing and/or intensity random effects, and the corresponding fixed effects were also omitted. Interactions between sex and PS were explored by analyzing the sexes combined and estimating the fixed main effects and interaction of sex and PS for each of the random effects in the model. The age at peak height velocity was obtained as the age when the first derivative of the mean height curve was maximal, with its standard error estimated with the bootstrap.

A *P* value of 0.05 was used as the threshold for significance. The main analyses involved testing a number of hypotheses to generate the SITAR coefficients. No adjustments were made for the multiple testing, and this should be considered when interpreting the findings.

## Results

**FIGURE 1:**
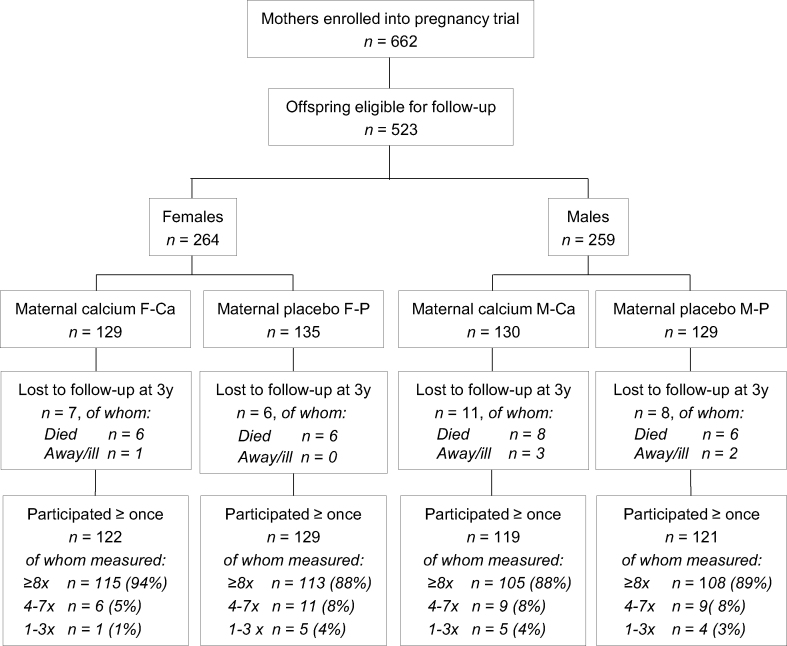
Flow of participants from the Gambian pregnancy calcium supplementation trial who were recruited at age 3 y into follow-up studies during childhood and adolescence. F-Ca, females born to mothers in the pregnancy calcium supplement group; F-P, females born to mothers in the pregnancy placebo group; M-Ca, males born to mothers in the pregnancy calcium supplement group; M-P, males born to mothers in the pregnancy placebo group.

The participants’ flow through the study is documented in Figure 1. A total of 491 of the 523 children eligible for the follow-up study (94%) were traced and enrolled. Of the remaining 32 children, 26 had died before 3 y of age, and 6 were either too unwell or lived too far away to participate. There were no significant differences between the 4 sex-PS groups in the numbers of children enrolled or lost to follow-up (Figure 1) or in maternal blood pressure, anthropometry, or other characteristics during pregnancy at 20 wk, prior to the start of supplementation, or at 36 wk of gestation (Supplemental Table 3). Most participants (90%) had blood pressure measurements and anthropometry at 8 or more of the 13 follow-up sessions (Figure 1). There were 39 (11%) of the 491 participants who did not attend a measurement session after the age of 15 y. The reasons for this drop-out were death (*n* = 7), illness (*n* = 1), away from home/not located (*n* = 26), and withdrawal from the cohort (*n* = 5). There was no evidence of a difference between the 4 sex-PS groups in number of deaths after age 3 y or other reasons for nonattendance after age 15 y.

Mothers of female participants had been lighter during pregnancy than mothers of male participants (mean ± SE female compared with male: 20 wk −1.5 ± 0.6 kg, *P* = 0.02; 36 wk −1.5 ± 0.7 kg, *P* = 0.03). There were no significant PS effects on children’s weight and length during infancy (Supplemental Table 4), and female participants had been significantly lighter and shorter than males throughout infancy (all sex differences *P* < 0.0001). Females had also gained less weight and length between 2 and 52 wk of age (*P* < 0.0001), although both sexes had experienced substantial growth faltering during infancy as shown by the lower (i.e., more negative) weight and length SD-scores at 52 wk (Supplemental Table 4).

### Cross-sectional comparisons

The participants’ blood pressure and anthropometry data at each timepoint are summarized by PS group for: age, SBP, and DBP in TABLE 1, TABLE 2 for females and males, respectively; height, weight, and BMI in TABLE 3, TABLE 4; the other anthropometry in Supplemental Tables 5 and 6; and pubertal status and dietary data assessed in the PSC series in Supplemental Tables 7 and 8**.**

**TABLE 1:** Age and blood pressure of female participants at each timepoint by pregnancy supplement group

Study	Age (y)				SBP (mmHg)				DBP (mmHg)			
	F-Ca		F-P		F-Ca		F-P		F-Ca		F-P	
	Mean ± SD	*n*	Mean ± SD	*n*	Mean ± SD	*n*	Mean ± SD	*n*	Mean ± SD	*n*	Mean ± SD	*n*
PSO.Y3	3.1 ± 0.1	122	3.0 ± 0.1	128	89.4 ± 10.1^1^	119	91.8 ± 9.4	126	53.6 ± 8.4	119	54.2 ± 9.7	126
PSO.Y5	5.0 ± 0.0	118	5.0 ± 0.1	124	90.9 ± 10.5	116	93.1 ± 10.2	121	58.0 ± 8.3	116	57.6 ± 7.8	121
PSO.Y7	7.0 ± 0.1	120	7.0 ± 0.1	116	97.5 ± 9.0	119	97.9 ± 9.4	116	60.1 ± 7.5	119	59.8 ± 6.8	116
PSE.B	7.4 ± 1.2	99	7.3 ± 1.2	97	97.4 ± 8.6	98	98.8 ± 9.7	97	58.2 ± 8.0	98	58.1 ± 7.5	97
PSO.Y9	9.0 ± 0.1	119	9.0 ± 0.1	119	102.4 ± 10.1	119	103.5 ± 9.4	119	61.8 ± 8.6	119	63.0 ± 7.2	119
PSC.B	9.2 ± 0.9	114	9.2 ± 0.9	117	—	—	—	—	—	—	—	—
PSO.Y11	11.0 ± 0.2	110	11.0 ± 0.1	114	102.2 ± 9.0	107	104.5 ± 9.3	112	61.1 ± 8.1	107	61.8 ± 7.3	112
PSO.Y13	13.0 ± 0.2	40	13.0 ± 0.1	36	104.7 ± 9.2	40	108.7 ± 11.7	36	65.2 ± 9.9	40	65.3 ± 8.7	36
PSC.F1	13.7 ± 1.3	104	13.8 ± 1.3	107	105.6 ± 9.9^2^	104	110.1 ± 11.4	107	60.8 ± 8.6^1^	104	63.6 ± 9.3	107
PSO.Y15	15.0 ± 0.1	85	15.0 ± 0.2	88	112.1 ± 8.9	84	114.4 ± 10.1	86	65.8 ± 8.2	85	67.4 ± 8.4	87
PSC.F2	16.3 ± 1.2	98	16.2 ± 1.3	99	111.1 ± 8.9	92	113.1 ± 9.6	97	63.7 ± 7.3^1^	92	65.9 ± 7.1	97
PSO.Y17	17.0 ± 0.0	44	17.0 ± 0.1	46	116.8 ± 8.9	39	118.1 ± 8.3	45	67.7 ± 8.9	43	68.8 ± 7.4	46
PSC.F3	18.4 ± 1.2	94	18.5 ± 1.5	94	113.5 ± 8.3	93	114.9 ± 10.4	93	69.7 ± 6.9	93	69.0 ± 8.1	93

Blood pressure was measured using Dinamap 8100 in the PSO series and Omron 7051T in PSE.B and the PSC series. Blood pressure was not measured at timepoint PSC.B.

Significance of difference in cross-sectional analysis between female offspring of mothers in the pregnancy supplement groups using analysis of covariance with age adjustment.

^1^*P* < 0.05.

^2^*P* = 0.001.

Abbreviations: DBP, diastolic blood pressure, F-Ca, females born to mothers in the pregnancy calcium supplement group; F-P, females born to mothers in the pregnancy placebo group; PSC.B to PSC.F3, Pregnancy Study Children’s Bone Health, Baseline, and Follow-up Studies; PSE.B, Pregnancy Study Early Nutrition Project; cohort B; PSO.Y3 to PSO.Y17, Pregnancy Study Offspring Study at timepoints Y3 to Y17; SBP, systolic blood pressure.

**TABLE 2:** Age and blood pressure of male participants at each timepoint by pregnancy supplement group

Study	Age (years)				SBP (mmHg)				DBP (mmHg)			
	M-Ca		M-P		M-Ca		M-P		M-Ca		M-P	
	Mean ± SD	*n*	Mean ± SD	*n*	Mean ± SD	*n*	Mean ± SD	*n*	Mean ± SD	*n*	Mean ± SD	*n*
PSO.Y3	3.1 ± 0.2	117	3.1 ± 0.2	121	89.9 ± 10.3	115	91.8 ± 9.5	119	53.2 ± 9.0	115	53.7 ± 9.0	119
PSO.Y5	5.0 ± 0.0	115	5.0 ± 0.1	118	90.6 ± 9.8	109	90.9 ± 9.1	114	55.0 ± 7.4	109	56.8 ± 7.4	114
PSO.Y7	7.0 ± 0.0	110	7.0 ± 0.1	117	96.0 ± 8.9	109	95.4 ± 9.4	116	57.5 ± 7.6	110	58.0 ± 7.6	116
PSE.B	7.5 ± 1.2	94	7.4 ± 1.2	99	98.1 ± 8.2	94	96.7 ± 7.9	99	56.9 ± 7.4	94	56.3 ± 7.2	99
PSO.Y9	9.0 ± 0.1	111	9.0 ± 0.1	114	99.0 ± 8.0	110	99.6 ± 7.7	114	59.2 ± 7.1	111	60.7 ± 6.8	114
PSC.B	9.2 ± 0.9	109	9.3 ± 0.9	107	—	—	—	—	—	—	—	—
PSO.Y11	11.0 ± 0.1	101	11.0 ± 0.2	105	100.5 ± 8.7^1^	98	98.2 ± 8.0	103	60.2 ± 7.8	98	59.0 ± 7.3	102
PSO.Y13	13.0 ± 0.1	31	13.1 ± 0.2	28	99.4 ± 8.6	31	99.9 ± 6.7	28	60.6 ± 7.7	31	59.6 ± 7.6	28
PSC.F1	13.9 ± 1.2	89	13.9 ± 1.2	98	103.7 ± 10.9	88	102.5 ± 11.6	98	58.8 ± 9.7	88	58.1 ± 9.6	98
PSO.Y15	15.0 ± 0.1	75	15.0 ± 0.0	80	109.5 ± 11.5	75	108.9 ± 10.4	80	63.4 ± 7.1	75	62.9 ± 8.2	80
PSC.F2	16.3 ± 1.3	89	16.3 ± 1.2	92	111.5 ± 11.1	88	109.8 ± 11.1	91	61.7 ± 8.6	88	60.6 ± 8.5	91
PSO.Y17	17.0 ± 0.0	40	17.0 ± 0.1	40	116.4 ± 11.1	39	115.7 ± 9.5	40	64.0 ± 7.0	39	65.3 ± 7.1	40
PSC.F3	18.4 ± 1.5	80	18.3 ± 1.3	84	117.5 ± 10.2	79	117.8 ± 10.3	84	67.7 ± 8.3	79	67.0 ± 8.3	84

Blood pressure was measured using Dinamap 8100 in the PSO series and Omron 7051T in PSE.B and the PSC series. Blood pressure was not measured at timepoint PSC.B.

Significance of difference in cross-sectional analysis between male offspring of mothers in the pregnancy supplement groups using analysis of covariance with age adjustment.

^1^*P* = 0.05.

Abbreviations: DBP, diastolic blood pressure; M-Ca, males born to mothers in the pregnancy calcium supplement group; M-P; males born to mothers in the pregnancy placebo group; PSC.B to PSC.F3, Pregnancy Study Children’s Bone Health Baseline and Follow-up Studies; PSE.B, Pregnancy Study Early Nutrition Project; cohort B; PSO.Y3 to PSO.Y17, Pregnancy Study Offspring Study at timepoints Y3 to Y17; SBP, systolic blood pressure.

**TABLE 3:** Height, weight, and BMI of female participants at each timepoint by pregnancy supplement group

Study	Height (cm)				Weight (kg)				BMI (kg/m^2^)			
	F-Ca		F-P		F-Ca		F-P		F-Ca		F-P	
	Mean ± SD	*n*	Mean ± SD	*n*	Mean ± SD	*n*	Mean ± SD	*n*	Mean ± SD	*n*	Mean ± SD	*n*
PSO.Y3	87.8 ± 3.6	121	87.9 ± 3.9	125	11.8 ± 1.3	121	11.9 ± 1.3	125	15.3 ± 1.4	121	15.4 ± 1.5	124
PSO.Y5	102.3 ± 4.2	116	102.7 ± 4.6	121	15.0 ± 1.7	117	15.2 ± 1.9	122	14.4 ± 1.4	115	14.4 ± 1.3	121
PSO.Y7	115.2 ± 4.2	112	115.6 ± 5.2	111	18.8 ± 2.1	118	18.8 ± 2.4	114	14.1 ± 1.1	111	14.1 ± 1.2	110
PSE.B	118.0 ± 7.8	99	118.5 ± 8.4	97	19.3 ± 3.2	99	19.8 ± 3.9	97	13.8 ± 1.2	99	13.9 ± 1.2	97
PSO.Y9	126.8 ± 4.7	113	127.2 ± 5.6	113	23.2 ± 2.8	116	23.9 ± 3.8	117	14.5 ± 1.2	112	14.7 ± 1.5	112
PSC.B	127.6 ± 6.6^1^	114	128.8 ± 6.7	115	23.6 ± 3.5	114	24.4 ± 4.4	117	14.4 ± 1.2	114	14.5 ± 1.4	115
PSO.Y11	137.4 ± 5.4	109	138.4 ± 6.8	112	29.3 ± 3.9	109	30.0 ± 5.6	113	15.5 ± 1.5	109	15.6 ± 2.1	111
PSO.Y13	148.9 ± 7.3	39	152.0 ± 7.7	36	35.9 ± 6.5	39	38.1 ± 7.7	36	16.1 ± 1.9	39	16.3 ± 2.1	36
PSC.F1	151.5 ± 7.7^2^	104	153.7 ± 8.5	107	39.1 ± 8.1^2^	104	42.0 ± 10.3	107	16.9 ± 2.3	104	17.5 ± 3.0	107
PSO.Y15	156.7 ± 6.0	83	157.6 ± 6.3	85	46.1 ± 8.1	83	47.7 ± 8.6	87	18.7 ± 2.7	83	19.0 ± 2.8	85
PSC.F2	158.9 ± 6.0	98	160.1 ± 6.0	99	48.6 ± 7.9	98	50.5 ± 10.4	99	19.2 ± 2.8	98	19.6 ± 3.4	99
PSO.Y17	159.1 ± 5.6	42	159.4 ± 5.8	46	50.6 ± 6.7	43	54.1 ± 10.2	46	19.9 ± 2.4^1^	42	21.3 ± 3.7	46
PSC.F3	160.5 ± 5.8	94	161.1 ± 5.4	94	52.9 ± 7.4	94	53.9 ± 8.7	94	20.5 ± 2.8	94	20.8 ± 3.3	94

Significance of difference in cross-sectional analysis between female offspring of mothers in the pregnancy supplement groups using analysis of covariance with age adjustment.

^1^*P* < 0.05.

^2^*P* = 0.01.

Abbreviations: F-Ca, females born to mothers in the pregnancy calcium supplement group; F-P, females born to mothers in the pregnancy placebo group; PSC.B to PSC.F3, Pregnancy Study Children’s Bone Health, Baseline and Follow-up Studies; PSE.B, Pregnancy Study Early Nutrition Project; cohort B; PSO.Y3 to PSO.Y17, Pregnancy Study Offspring Study at timepoints Y3 to Y17.

**TABLE 4:** Height, weight, and BMI of male participants at each timepoint by pregnancy supplement group

Study	Height (cm)				Weight (kg)				BMI (kg/m^2^)			
	M-Ca		M-P		M-Ca		M-P		M-Ca		M-P	
	Mean ± SD	*n*	Mean ± SD	*n*	Mean ± SD	*n*	Mean ± SD	*n*	Mean ± SD	*n*	Mean ± SD	*n*
PSO.Y3	88.7 ± 3.6	114	88.6 ± 3.5	118	12.3 ± 1.5	113	12.4 ± 1.2	120	15.7 ± 1.7	113	15.8 ± 1.3	118
PSO.Y5	103.0 ± 4.5	111	103.2 ± 4.1	112	15.5 ± 1.9	114	15.4 ± 1.6	117	14.5 ± 1.3	111	14.4 ± 1.2	112
PSO.Y7	115.2 ± 4.6	105	115.6 ± 4.6	111	19.0 ± 2.3	110	18.9 ± 1.9	116	14.3 ± 1.1	105	14.2 ± 1.1	111
PSE.B	118.2 ± 7.5	94	118.3 ± 7.9	98	20.1 ± 3.4	94	19.7 ± 2.9	99	14.3 ± 1.1^1^	94	13.9 ± 0.9	98
PSO.Y9	126.1 ± 4.9	108	125.8 ± 4.7	114	23.3 ± 2.9	111	22.9 ± 2.6	114	14.7 ± 1.2	108	14.5 ± 1.2	114
PSC.B	127.5 ± 6.5	109	127.3 ± 6.3	107	24.0 ± 3.7	109	23.6 ± 3.3	107	14.7 ± 1.2	109	14.5 ± 1.1	107
PSO.Y11	135.9 ± 6.0	100	135.7 ± 5.6	103	28.5 ± 3.9	101	28.2 ± 3.3	104	15.3 ± 1.4	100	15.3 ± 1.3	103
PSO.Y13	146.2 ± 6.9	31	146.0 ± 5.6	28	32.6 ± 5.0	31	32.9 ± 2.7	28	15.2 ± 1.5	31	15.5 ± 1.1	28
PSC.F1	149.7 ± 10.4	89	149.0 ± 10.4	98	36.6 ± 8.0	89	35.6 ± 7.3	98	16.1 ± 1.8	89	15.9 ± 1.5	98
PSO.Y15	155.0 ± 8.5	75	155.1 ± 8.2	77	40.4 ± 7.3	75	40.4 ± 7.1	79	16.7 ± 1.8	75	16.7 ± 1.8	77
PSC.F2	163.1 ± 10.0	89	162.5 ± 9.9	92	47.2 ± 9.9	89	45.7 ± 8.9	92	17.6 ± 2.1	89	17.2 ± 1.9	92
PSO.Y17	165.8 ± 7.1	38	165.3 ± 7.3	39	50.4 ± 8.6	39	48.2 ± 8.1	40	18.3 ± 2.2	38	17.7 ± 2.1	39
PSC.F3	170.1 ± 7.9	80	170.3 ± 8.2	84	56.2 ± 9.9	80	53.6 ± 8.5	84	19.3 ± 2.5^1^	80	18.4 ± 2.1	84

Significance of difference in cross-sectional analysis between male offspring of mothers in the pregnancy supplement groups using analysis of covariance with age adjustment.

^1^*P* < 0.05.

Abbreviations: BMI, body mass index; M-Ca, males born to mothers in the pregnancy calcium supplement group; M-P; males born to mothers in the pregnancy placebo group; PSC.B to PSC.F3, Pregnancy Study Children’s Bone Health, Baseline and Follow-up Studies; PSE.B, Pregnancy Study Early Nutrition Project; cohort B; PSO.Y3 to PSO.Y17, Pregnancy Study Offspring Study at timepoints Y3 to Y17; SD, standard deviation.

With the supplement groups combined, SBP and DBP tended to be higher in females than males, highly significantly so during later childhood and early adolescence, although the difference in SBP reduced at later timepoints and was lower than in males by 18 y (Supplemental Table 9). Height, weight, and BMI were independent predictors of SBP and DBP at each timepoint when considered separately, weight being the strongest predictor. They were also predictors of SBP but not DBP in males at most timepoints. Height was generally not significant when weight was also included in the model.

Females had similar heights and lower weights than males in childhood but were taller and heavier with higher BMI in early adolescence from ∼9 y. By late teenage, they were shorter than males with a higher BMI (Supplemental Table 9). They also had higher MUAC and TST at most ages (Supplemental Table 10). Relative to the UK reference data, both females and males had low height and weight for their age throughout childhood and adolescence, markedly so in their early years and, for males, during puberty (Supplemental Tables 11 and 12).

### Longitudinal comparisons

SITAR models were fitted to the longitudinal data, contrasting the shapes of the mean curves for the 2 PS groups by sex. The final models for height included size, timing, and intensity; those for weight, BMI, MUAC, and TST excluded timing to ensure the models converged and those for SBP, DBP, and HC excluded both timing and intensity for the same reason, leaving just size.

**FIGURE 2:**
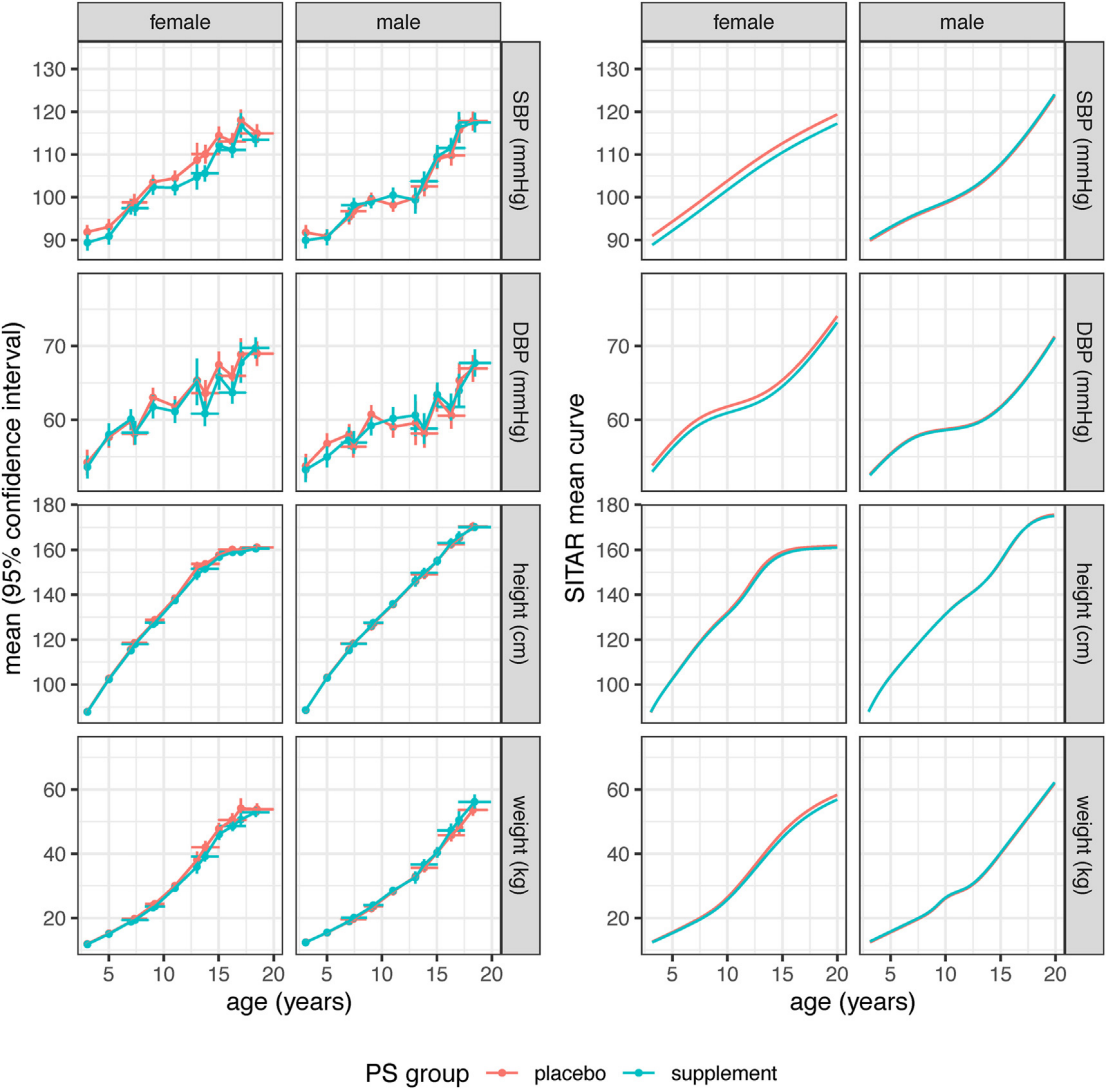
Blood pressure, height and weight during childhood and adolescence, by sex and PS group, of Gambian children whose mothers had participated in the pregnancy calcium supplement trial. The left half of the figure shows cross-sectional mean values of SBP, DBP, height and weight by PS group, sex and age for each distinct measurement occasion, where the y-bars indicate the mean ± 95% confidence interval, whereas the *x*-bars show mean age ± 1 SD. The right half of the figure shows the corresponding mean SITAR curves, generated using the SITAR mixed effects growth curve model [29]. These summarize the same data, with separate curves for the Ca (blue) and P (red) groups by sex. The blood pressure models included an adjustment for the 2 different instruments used. DBP, diastolic blood pressure; PS group, pregnancy supplement group dividing offspring into those whose mothers were in the calcium supplement group during pregnancy (Ca) and those whose mothers were in the placebo group (P); SBP, systolic blood pressure.

**FIGURE 3:**
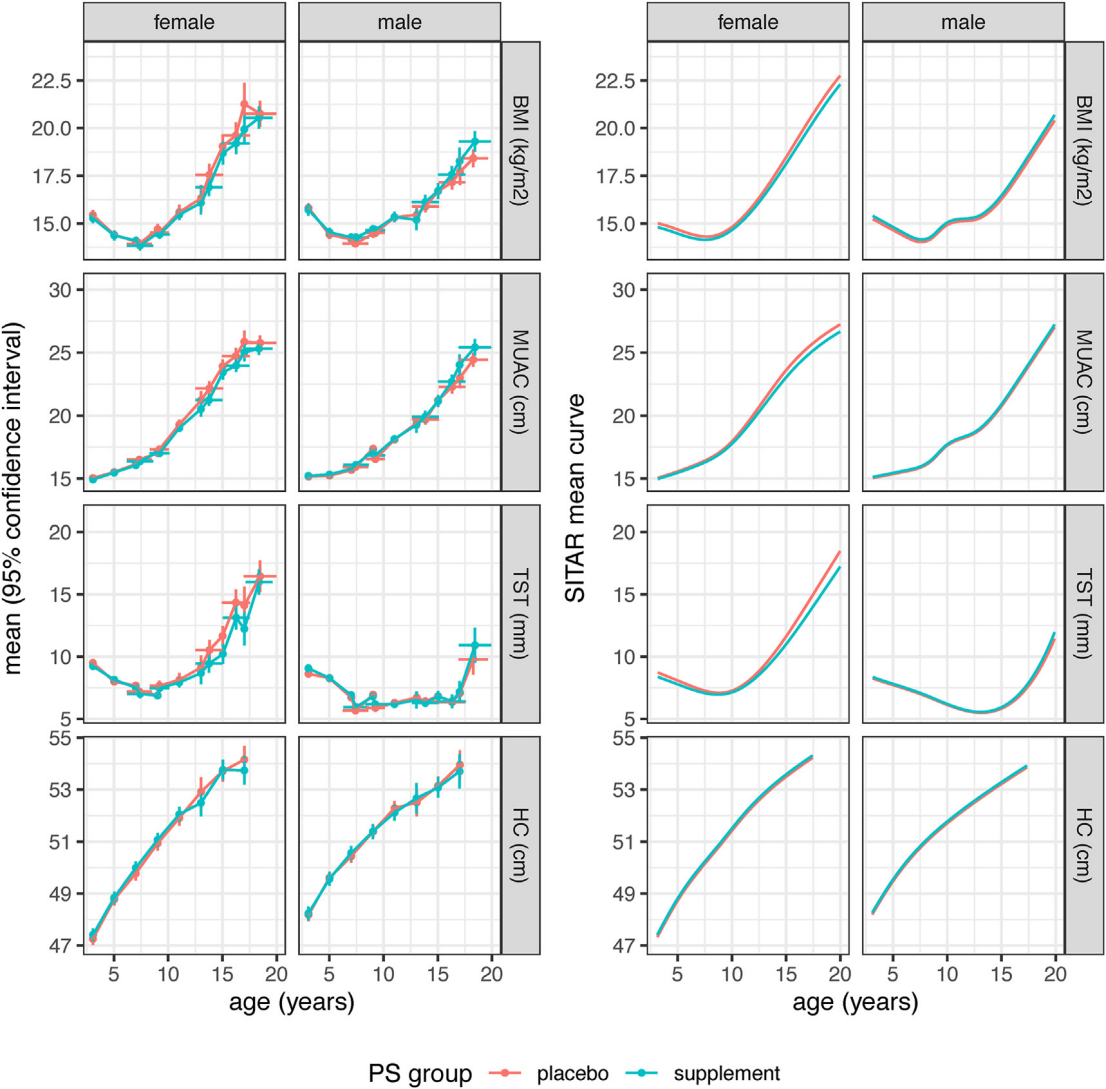
BMI, mid-upper arm circumference, triceps skinfold thickness and head circumference during childhood and adolescence, by sex and PS group, of Gambian children whose mothers had participated in a pregnancy calcium supplement trial. The left half of the figure shows cross-sectional mean values of BMI, MUAC, TST, and HC by PS group, sex and age for each distinct measurement occasion, where the y-bars indicate the mean ± 95% confidence interval, whereas the *x*-bars show mean age ± 1 SD. The right half of the figure shows the corresponding mean SITAR curves, generated using the SITAR mixed effects growth curve model [29]. These summarize the same data, with separate curves for the Ca (blue) and P (red) groups by sex. HC, head circumference; MUAC, mid-upper arm circumference; PS group, pregnancy supplement group categorizing offspring into those whose mothers were in the calcium supplement group during pregnancy (Ca) and those whose mothers were in the placebo group (P); SITAR, SuperImposition by Translation and Rotation; TST, triceps skinfold thickness.

FIGURE 2, FIGURE 3 compare the Ca and P mean curves for each outcome visually by sex, with the raw data to the left and the fitted SITAR mean curves to the right. Figure 2 covers SBP, DBP, height, and weight, whereas Figure 3 includes BMI, MUAC, TST, and HC. Figure 2 shows that in females, but not males, the Ca curves for SBP and DBP were appreciably lower than the P curves, corresponding to differences in SITAR size, i.e., the intercept, between Ca and P. FIGURE 2, FIGURE 3 also show that in females, though not males, the curves for other outcomes tended to be shallower for Ca than P, i.e., negative intensity. For males, the pairs of curves were so similar as to be superimposed in some cases.

**FIGURE 4:**
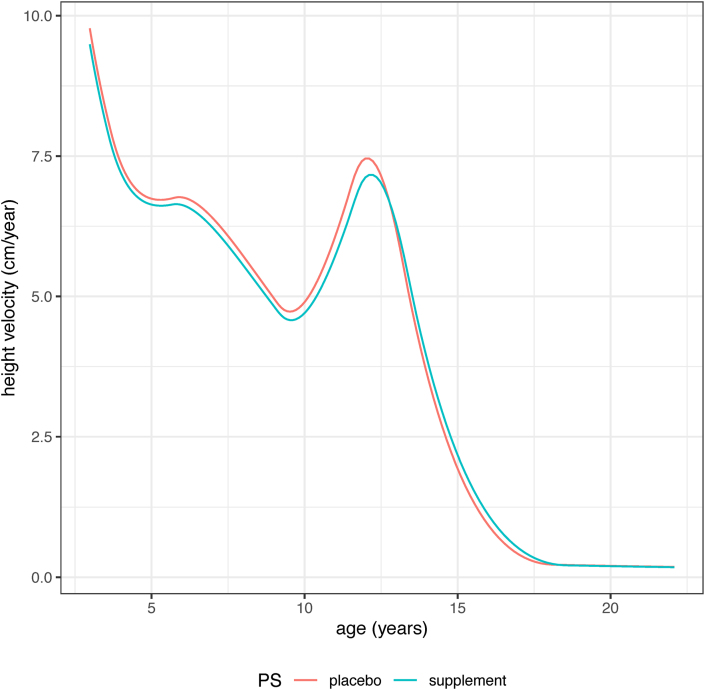
Mean SITAR height velocity curves by PS group for Gambian females whose mothers had participated in a pregnancy calcium supplement trial. Curves were generated using the SITAR mixed effects growth curve model [29] and show that the mean velocity was significantly lower in the Ca (blue) than the P (red) group (Ca vs. *P* = −2.9 ± SE 1.1%, *P* = 0.009). PS group, pregnancy supplement group categorizing offspring into those whose mothers were in the calcium supplement group during pregnancy (Ca) and those whose mothers were in the placebo group (P); SE, standard error; SITAR, SuperImposition by Translation and Rotation.

Table 5 summarizes the SITAR results comparing the Ca and P curves for the different outcomes by sex. For females, the SBP size difference (F-Ca compared with F-P) of −2.1 ± 0.8 mmHg was highly significant (*P* = 0.005), whereas the DBP difference of −0.9 ± 0.6 mmHg was not (*P* = 0.2). There was also a significant difference in SITAR intensity for height in females, smaller for the Ca group at −2.9 ± 1.1% (*P* = 0.009), corresponding to the Ca curve being shallower and peak height velocity 3% smaller (Figure 4). Intensity was also less for all the female outcomes where intensity was fitted, meaning that the Ca curves were shallower than the P curves, although apart from height, none were significant (Table 5). For males, there were no consistent differences in the Ca and P mean curves for any outcome.

**TABLE 5:** Blood pressure and anthropometry of participants: SITAR contrasts comparing means for the pregnancy supplement groups by sex

Measure (units)	SITAR size (units)		SITAR timing (%)		SITAR intensity (%)	
	Ca vs. P ± SE	*P*	Ca vs. P ± SE	*P*	Ca vs. P ± SE	*P*
Females						
SBP (mmHg)	−2.13 ± 0.77	0.005^1^	—	—	—	—
DBP (mmHg)	−0.86 ± 0.60	0.2	—	—	—	—
Height (cm)	−0.69 ± 0.71	0.3	0.2 ± 1.1	0.9	−2.9 ± 1.1	0.009
Weight (kg)	−0.75 ± 0.48	0.1	—	—	−5.2 ± 3.3	0.1
BMI (kg/m^2^)	−0.16 ± 0.15	0.3	—	—	−4.4 ± 5.4	0.4
MUAC (cm)	−0.22 ± 0.15	0.1	—	—	−7.3 ± 4.0	0.06
TST (mm)	−0.14 ± 0.14	0.3	—	—	−8.7 ± 6.0	0.1
HC (cm)	0.09 ± 0.16	0.6	—	—	—	—
Males						
SBP (mmHg)	0.35 ± 0.78	0.6^1^	—	—	—	—
DBP (mmHg)	−0.14 ± 0.58	0.8	—	—	—	—
Height (cm)	−0.68 ± 1.00	0.5	−1.2 ± 1.5	0.4	−0.8 ± 1.5	0.6
Weight (kg)	0.29 ± 0.38	0.4	—	—	0.2 ± 2.4	0.9
BMI (kg/m^2^)	0.13 ± 0.13	0.3	—	—	1.9 ± 3.0	0.5
MUAC (cm)	0.10 ± 0.12	0.4	—	—	1.0 ± 2.7	0.7
TST (mm)	0.06 ± 0.13	0.6	—	—	2.2 ± 5.4	0.7
HC (cm)	0.07 ± 0.17	0.7	—	—	—	—

Data are mean ± SE, *P* value, for the comparison of the calcium supplement group with the placebo group, obtained using the SITAR mixed effects growth curve model [29]. Models that failed to converge were simplified by omitting the timing and/or intensity random effects, indicated in the table by dashes. Models for SBP and DBP included an adjustment to account for differences between the 2 blood pressure instruments used.

Significance of pregnancy group by sex interaction: ^1^regression coefficient = −2.5, SE = 1.1, *P* = 0.02. No other interactions were significant.

Abbreviations: Ca, offspring of mothers supplemented with calcium during pregnancy; DBP, diastolic blood pressure; HC, head circumference; P, offspring of mothers in the placebo group during pregnancy; MUAC, mid-upper arm circumference; SITAR, SuperImposition by Translation and Rotation; SBP, systolic blood pressure; SE, standard error; TST, triceps skinfold thickness.

Analyzing the blood pressure data with the sexes combined showed that SBP was higher in females than males, by 3.2 ± SE 0.8 mmHg (*P* < 0.0001), and there was a significant Ca-P by sex interaction (*P* = 0.02), indicating that the Ca-P effect was greater in females than males. Similarly, DBP was higher in females than that in males, by 2.7 ± SE 0.6 mmHg (*P* < 0.0001), although the Ca-P by sex interaction was not significant (*P* = 0.4).

### Pubertal development and dietary intake

The mean age of peak height velocity was 12.1 ± SE 0.1 y for females and 15.3 ± SE 0.1 y for males. By mean age 18.4 y, the majority of both sexes had not yet reached stage 5 (Supplemental Table 7). There was no significant difference in mean ± SE age at peak height velocity between Ca and P groups in either sex (F-Ca compared with F-P: 0.0 ± SE 0.1 y; *P* = 0.9; M-Ca compared with M-P: −0.2 ± SE 0.2 y; *P* = 0.4). The age of menarche in those females whose menstruation had started by their last measurement session (86%, 215/251) was mean ± SD = 14.7 ± 1.2 y, median (IQR) = 14.7 (13.8–15.5) y, and there was no significant difference between the PS groups (F-Ca compared with F-P: 0.11 ± 0.17 y; *P* = 0.5). The calcium intakes in both sexes were low throughout late childhood and adolescence, with mean values ∼300 mg/d, and there were no apparent differences between the PS groups in dietary intakes in either sex (Supplemental Table 8).

## Discussion

This intensive longitudinal study revealed long-term effects of pregnancy calcium supplementation in a population with a low-calcium intake on offspring blood pressure and growth that differed between the sexes.

In many parts of the world, calcium intake during pregnancy is low compared to international recommendations, especially in low- and middle-income countries [30]. Calcium is needed in pregnancy for the growth of the fetal skeleton and for many other facets of fetal and maternal health. Although there are physiologic mechanisms that act to ensure an adequate calcium supply when calcium requirements increase [31], there are concerns that a low-calcium intake during pregnancy may be limiting, increasing the risk of gestational hypertension, pre-eclampsia, and poor growth of the fetus [1].

The WHO recommends calcium supplementation at 1500–2000 mg Ca/d for pregnant women in populations with a low-calcium intake to reduce the risk of pre-eclampsia and its complications [1]. A recent systematic review and meta-analysis has reaffirmed the WHO position, showing a reduction in pre-eclampsia risk and other poor pregnancy outcomes by calcium supplementation when calcium intake is low [32]. In that article, <900 mg/d was the criterion for a low-calcium intake, and lower supplementation doses (<1000 mg Ca/d) were shown to be as effective for pre-eclampsia prevention as higher doses (≥1000 mg/d) [32].

Few of the trials conducted in pregnancy and included in these published systematic reviews and meta-analyses incorporated any long-term follow-up of the health of the mother or her offspring following pregnancy calcium supplementation. Two trials reported effects on offspring blood pressure: in an Argentinian study, a lower mean SBP of 1.4 mmHg was recorded at age 5–9 y in offspring of mothers supplemented with 2000 mg Ca/d, an effect that was particularly pronounced among overweight children [3], and in a trial in the United States, where mothers were also supplemented with 2000 mg Ca/d, a lower mean SBP of 4.8 mmHg was recorded in their offspring at age 2 y [2].

The data presented in this paper were from a third trial that included long-term follow-up of both the mother and the offspring. This trial was conducted in a rural area of The Gambia, West Africa, where the customary calcium intake is low (300–400 mg/d). Trial participants were supplemented with 1500 mg Ca/d or placebo from 20 wk of pregnancy to term [17,18]. No short-term effects of the pregnancy calcium supplement were observed on maternal blood pressure or on fetal and infant growth [17,18], but unexpected longer-term effects were found. For the mothers, lactational bone mineral mobilization was greater among those supplemented with calcium in pregnancy compared with placebo, and only partial skeletal restitution had occurred in the calcium-supplemented group after 5 y [33]. For the offspring, there was evidence at age 8–12 y that the maternal supplement had altered childhood growth trajectories and bone development such that female offspring of mothers who had been calcium supplemented (F-Ca) had slower growth compared with the placebo group (F-P), whereas male offspring of calcium-supplemented mothers (M-Ca) had accelerated growth [20]. These sex effects of maternal calcium supplementation were also seen in blood samples collected at mean age 7 y in which plasma insulin-like growth factor-1 (IGF1) concentrations were significantly lower in F-Ca than F-P but higher in M-Ca than M-P [23].

The analysis reported here of measurements recorded longitudinally among the offspring of the mothers in the Gambian trial from age 3 y to young adult life has confirmed that the maternal supplement had long-term effects on the blood pressure and growth of the children that differed between the sexes. Throughout childhood and adolescence, F-Ca had lower mean SBP than F-P by 2 mmHg. This difference is similar to those reported in young offspring after the Argentinian and United States pregnancy trials. Blood pressure is known to track from childhood into adult life [4], and a lowering of a population mean blood pressure by as little as 2–3 mmHg is comparable with the benefits achieved in adults through dietary interventions and other means [34,35]. This could be of importance in a country, such as The Gambia, where rates of adult hypertension and stroke are high, even among resource-poor communities in rural areas [13,14].

The effect of pregnancy calcium supplementation on growth in female offspring was most evident in differences in the shape of the height velocity curve. This was evidenced by the significant SITAR intensity effect indicating a shallower curve and a 3% lower peak height velocity in F-Ca than F-P. This can be interpreted as developmental time being stretched relative to chronologic time in F-Ca, which extends the period of the growth spurt and reduces mean velocity (Figure 4). Similar differences in the patterns of growth were seen in weight and the other anthropometry, although none of their SITAR effects was significant. Although no significant differences were evident in the assessments of pubertal development or age of menarche, the different patterns of growth in female offspring resulting from the maternal calcium supplementation could have societal and health consequences, beneficial or otherwise, which requires additional research to explore further.

In clear contrast to the results for females, for males, there were no differences in growth pattern for blood pressure or anthropometry, to the extent that their mean curves in FIGURE 2, FIGURE 3 are in most cases superimposed. This is persuasive evidence of sexual dimorphism in the offspring response to maternal supplementation.

Taken together with previous observations in this Gambian cohort, it is clear that pregnancy supplementation with high-dose calcium when maternal calcium intake is low leads to effects on offspring blood pressure and growth that differ between females and males. The underlying mechanisms are unknown but may relate to alterations in the fetal programming of the growth hormone–IGF1 axis. The potential for in utero IGF1 programming by maternal calcium intake has been demonstrated in trials of milk supplementation [36], observational studies of maternal milk consumption [37], and animal studies [38]. The possibility of differential effects between female and male offspring was not examined in many of these studies, but sex differences have been reported in the response to maternal diet [39], micronutrient supplementation [40], and famine [41] and to cigarette use and asthma in pregnancy [42]. As with the Gambian cohort [23], many of these reports describe corresponding effects on IGFs, their binding proteins, and other related growth factors.

Our study has many strengths, most notably the high-retention rate with 90% of children measured on 8 or more occasions from 3 y to early adulthood, and the balanced numbers in the groups at each age, mirroring the original trial randomization. In addition, SITAR is robust to missing data and drop-out. It is limited by the use of 2 blood pressure instruments from different manufacturers, necessitated by the logistics of the studies, that consistently differed in their blood pressure readings, although this was adjusted for. It is also limited by the noise in the longitudinal data, as evidenced for example by the SITAR height models, where the residual SDs of 1.8 cm for females and 1.5 cm for males were about twice as large as those seen in other studies fitting SITAR models [29], which may have reduced the ability to detect significant differences. This may be due to factors, such as seasonality or to the inherent challenges in community-based follow-up studies in rural Africa.

This study showed, in female offspring, that pregnancy calcium supplementation in a population with a low-customary calcium intake may lower SBP and slow linear growth in childhood and adolescence, adding to evidence of offspring sexual dimorphism in responses to maternal supplementation. It adds to the previous reports in this cohort of effects on IGF1 and bone development that were different, and in opposite directions, between female and male offspring [20,23]. Although the lower blood pressure of females whose mothers received calcium supplementation in pregnancy may be beneficial for their long-term health, the consequences of different patterns of growth and bone development for both sexes are unknown. In addition, the observed effect of pregnancy calcium supplementation on maternal bone mineral density [33] could affect skeletal health into and after menopause. Further research is warranted on the long-term and intergenerational effects of antenatal supplementation.

## Acknowledgments

We thank the participants and their families for their steadfast commitment to these studies. We acknowledge the involvement of facilities at the MRC Unit The Gambia @ London School of Tropical Medicine and Hygiene (formerly MRC Keneba, The Gambia) and the MRC Elsie Widdowson Laboratory (formerly MRC Human Nutrition Research). In particular, we acknowledge the contributions of the Gambian research staff involved in primary data collection, logistics, and management, especially: Fabakary Bajo, Isatou Camara, Buba Ceesay, Kabiru Ceesay, Lamin Jammeh, Mariama Jammeh, Ebou Jarjou, Sheriff Kolley, Michael Mendy, Morikebba Sanyang, Yankuba Sawo, and late Saul Jarjou and Fatou Manneh. We also acknowledge Dr Sophie Hawkesworth, who conducted the PSE.B study while at the London School of Tropical Medicine and Hygiene.

## Author contributions

The authors’ responsibilities were as follows – AP, LMAJ, TJC: conceived the project plan for the data analysis in this paper; AP, LMAJ: conceived the original trial and offspring follow-up studies; AP, LMAJ, SEM, KAW: conducted the offspring follow-up studies and were responsible for the archived data; AP, LMAJ, SMS, TJC: collated the data and conducted the statistical analyses; AP, TJC: wrote the paper; AP: has primary responsibility for the content; and all authors: read and approved the final manuscript.

## Conflict of interest

KAW received honoraria from Abbott Nutrition for giving educational lectures on nutrition and growth unrelated to the work presented in this paper. All other authors report no conflicts of interest.

## Funding

Supported by UKRI Medical Research Council (MRC) under programs U105960371, U123261351, and MCA760-5QX00, and the Department for International Development (DFID) under the MRC/DFID Concordat agreement. The measurements made during the “Early Nutrition Project” (FOOD-CT-2005-007036) were also supported by the European Union Sixth Framework Programme for Research and Technical Development of the European Union Community.

## Data sharing statement

Data described in the manuscript will be made available upon reasonable request to the corresponding author and after clearance by the Scientific Co-ordinating and Ethics Committees of the MRC Unit The Gambia.
