# Supplementary material for: Effects of maternal calcium supplementation on offspring blood pressure and growth in childhood and adolescence in a population with a low-calcium intake: follow-up study of a randomized controlled trial
Source: Am J Clin Nutr. 2024 Apr 18;119(6):1443–54. doi: 10.1016/j.ajcnut.2024.02.025 (PMC11196864; doi:10.1016/j.ajcnut.2024.02.025)
Supplement: Multimedia component 1 [file mmc1.docx]

**Supplemental Table 1.** Study label, full title and ethics approvals

| Label | Full name | | Ethics Number* | Year approved | |  |
| --- | --- | --- | --- | --- | --- | --- |
|  |  | |  |  | |  |
| PSO.Y3-Y17 | Calcium requirements of pregnant Gambian women, the inclusion of blood pressure measurements on the children (PS Offspring Study with measurements every two years from 3 years of age) | SCC 585/599 | | | 1998 | |
| PSE.B | Long-term follow-up of health effects in offspring born following (A) protein-energy and (B) calcium supplementation trials during pregnancy (EARNEST Study) | SCC1013 | | | 2005 | |
| PSC.B | Bone status in 8-12 year old children whose mothers were supplemented with calcium during pregnancy (PS Child Baseline) | SCC1073 | | | 2007 | |
| PSC.F1-F3 | Bone status in 8-12 year old children whose mothers were supplemented with calcium during pregnancy – serial measurements of bone growth and development (PS Child Follow-up Studies) | L.2011.35 | | | 2011/12 | |

*Approvals given by The Gambia Government/MRC Laboratories Joint Ethics Committee

**Supplemental Table 2.** Calendar dates and participant ages of the follow-up studies

Schematic diagram illustrating the approximate range of calendar years and ages of the participants at each timepoint, not to scale.

Mean ± SD ages can be found in Supplemental Table 9.

*Abbreviations*: PSO.Y3 to PSO.Y17, Pregnancy Study Offspring Study at timepoints Y3 to Y17; PSE.B, Pregnancy Study Early Nutrition Project; cohort B; PSC.B to PSC.F3, Pregnancy Study Children’s Bone Health, Baseline and Follow-up Studies.

**Supplemental Table 3.** Maternal characteristics during pregnancy of participants in the follow-up studies

|  |  | | F-Ca | | | | |  | | F-P | | | |  | | M-Ca | | | | |  | | M-P | | | | | | | | | |
| --- | --- | --- | --- | --- | --- | --- | --- | --- | --- | --- | --- | --- | --- | --- | --- | --- | --- | --- | --- | --- | --- | --- | --- | --- | --- | --- | --- | --- | --- | --- | --- | --- |
|  |  | Mean±SD | | | *n* | |  | | Mean±SD | | *n* | |  | | Mean±SD | | | *n* | |  | | | | | Mean±SD | | | *n* | | | | |
| 20 weeks pregnancy | |  | |  |  | | |  | |  | |  | |  | |  | |  | | | | | | |  | | |  | | |  | |
| Age (y) | |  | | 28.1±6.9 | | | 76 | |  | | 27.8±6.9 | | 81 | |  | | 26.8±6.5 | | | 80 | | |  | | | | 27.6±7.3 | | | 77 | |  |
| Gravidity^1^ | |  | | 5 (3, 8) | | | 109 | |  | | 5 (3, 8) | | 115 | |  | | 5 (2, 7) | | | 107 | | |  | | | | 5 (2, 7) | | | 111 | |  |
| Parity^1^ | |  | | 4 (2, 6) | | | 118 | |  | | 4 (2, 6) | | 126 | |  | | 3 (2, 5) | | | 116 | | |  | | | | 4 (1, 5) | | | 120 | |  |
| SBP (mmHg) | |  | | 100.7±8.5 | | | 122 | |  | | 100.7±9.2 | | 129 | |  | | 101.6±8.7 | | | 119 | | |  | | | | 102.8±9.0 | | | 121 | |  |
| DBP (mmHg) | |  | | 54.5±7.7 | | | 122 | |  | | 55.1±8.0 | | 129 | |  | | 53.9±7.0 | | | 119 | | |  | | | | 56.3±7.6 | | | 121 | |  |
| Height (m) | |  | | 1.60±0.05 | | | 122 | |  | | 1.60±0.05 | | 129 | |  | | 1.60±0.05 | | | 119 | | |  | | | | 1.61±0.06 | | | 121 | |  |
| Weight (kg) | |  | | 54.6±7.1 | | | 122 | |  | | 54.0±7.1 | | 129 | |  | | 55.7±6.5 | | | 118 | | |  | | | | 55.9±7.2 | | | 120 | |  |
| BMI (kg/m^2^) | |  | | 21.3±2.3 | | | 122 | |  | | 21.2±2.4 | | 129 | |  | | 21.8±2.3 | | | 118 | | |  | | | | 21.6±2.6 | | | 120 | |  |
| 36 weeks pregnancy | |  | |  | | |  | |  | |  | |  | |  | |  | | |  | | |  | | | |  | | |  | |  |
| SBP (mmHg) | |  | | 102.6±8.7 | | | 121 | |  | | 103.4±8.7 | | 128 | |  | | 104.3±9.1 | | | 118 | | |  | | | | 106.2±11.4 | | | 121 | |  |
| DBP (mmHg) | |  | | 58.2±7.8 | | | 121 | |  | | 58.0±8.0 | | 128 | |  | | 58.4±8.6 | | | 118 | | |  | | | | 61.5±10.0 | | | 121 | |  |
| Weight (kg) | |  | | 58.9±7.3 | | | 120 | |  | | 58.3±7.3 | | 128 | |  | | 59.7±6.7 | | | 118 | | |  | | | | 60.2±7.7 | | | 121 | |  |

Data are for mothers of participants who were enrolled into the follow-up studies: F-Ca, females born to mothers in the pregnancy calcium supplement group; F-P, females born to mothers in the pregnancy placebo group; M-Ca, males born to mothers in the pregnancy calcium supplement group; M-P males born to mothers in the pregnancy placebo group. SBP, systolic blood pressure, DBP, diastolic blood pressure, BMI, body mass index. There were no significant differences between the pregnancy supplement groups of mothers with offspring of either sex. Blood pressure was measured using Dinamap 8100.

^1^Median (25,75 percentiles)

**Supplemental Table 4.** Early life characteristics of participants in the follow-up studies^1,2,3^

|  | |  | | F-Ca | | | |  | | F-P | | | |  | | M-Ca | | | |  | | M-P | | |  |
| --- | --- | --- | --- | --- | --- | --- | --- | --- | --- | --- | --- | --- | --- | --- | --- | --- | --- | --- | --- | --- | --- | --- | --- | --- | --- |
|  |  | | Mean±SD | | *n* | |  | | Mean±SD | | *n* | |  | | Mean±SD | | *n* | |  | | Mean±SD | | *n* | |  |
| Birth^1^ | |  | | |  | |  | |  | |  | |  | |  | |  | |  | |  | |  | |  |
| Gestational age (weeks) | |  | | | 38.1±1.5 | | 78 | |  | | 38.1±1.5 | | 86 | |  | | 38.6±1.6 | | 79 | |  | | 38.5±1.5 | | 88 |
| Length (cm) | |  | | | 48.4±2.1 | | 77 | |  | | 48.4±2.1 | | 86 | |  | | 48.7±2.8 | | 79 | |  | | 49.2±2.5 | | 89 |
| Weight (kg) | |  | | | 2.85±0.34 | | 77 | |  | | 2.82±0.35 | | 87 | |  | | 2.93±0.39 | | 79 | |  | | 3.06±0.40 | | 89 |
| Length (cm) | |  | | |  | |  | |  | |  | |  | |  | |  | |  | |  | |  | |  |
| 2 weeks | |  | | | 50.2±1.9 | | 120 | |  | | 50.4±1.9 | | 128 | |  | | 50.9±2.2 | | 117 | |  | | 51.3±2.1 | | 120 |
| 13 weeks | |  | | | 59.2±2.5 | | 120 | |  | | 59.0±2.7 | | 126 | |  | | 60.1±2.6 | | 118 | |  | | 60.6±2.4 | | 117 |
| 52 weeks | |  | | | 70.6±2.7 | | 118 | |  | | 70.5±3.1 | | 126 | |  | | 72.2±3.0 | | 116 | |  | | 72.7±3.4 | | 116 |
| Weight (kg) | |  | | |  | |  | |  | |  | |  | |  | |  | |  | |  | |  | |  |
| 2 weeks | |  | | | 3.23±0.41 | | 120 | |  | | 3.21±0.44 | | 128 | |  | | 3.42±0.59 | | 117 | |  | | 3.50±0.51 | | 120 |
| 13 weeks | |  | | | 5.55±0.72 | | 120 | |  | | 5.48±0.74 | | 126 | |  | | 6.02±0.84 | | 119 | |  | | 6.04±0.67 | | 119 |
| 52 weeks | |  | | | 7.62±0.99 | | 118 | |  | | 7.60±0.98 | | 126 | |  | | 8.24±1.16 | | 117 | |  | | 8.22±0.99 | | 116 |
| Length SDS | |  | | |  | |  | |  | |  | |  | |  | |  | |  | |  | |  | |  |
| 2 weeks | |  | | | -0.86±0.99 | | 120 | |  | | -0.74±1.02 | | 128 | |  | | -0.89±1.10 | | 117 | |  | | -0.67±1.04 | | 120 |
| 52 weeks | |  | | | -1.38±1.11 | | 118 | |  | | -1.40±1.29 | | 126 | |  | | -1.31±1.21 | | 116 | |  | | -1.12±1.34 | | 116 |
| Weight SDS | |  | | |  | |  | |  | |  | |  | |  | |  | |  | |  | |  | |  |
| 2 weeks | |  | | | -1.17±0.89 | | 120 | |  | | -1.21±0.94 | | 128 | |  | | -1.12±1.15 | | 117 | |  | | -0.95±1.00 | | 120 |
| 52 weeks | |  | | | -2.19±1.31 | | 118 | |  | | -2.21±1.27 | | 126 | |  | | -2.02±1.33 | | 117 | |  | | -2.03±1.16 | | 116 |

Data are for participants who were enrolled into the follow-up studies: F-Ca, females born to mothers in the pregnancy calcium supplement group; F-P, females born to mothers in the pregnancy placebo group; M-Ca, males born to mothers in the pregnancy calcium supplement group; M-P males born to mothers in the pregnancy placebo group; SDS, SD-score using the UK reference (Freeman JV *et al*, Archives of Disease in Childhood 1995;73:17-24) ;

^1^ Age of participants: Birth = measured within the first week of life, infancy (mean ± SD) 2 weeks = 2.1±0.9, 13 weeks = 13.7±1.8; 52 weeks = 52.8±1.3. There were no significant age differences between the groups.

^2^ There were no significant differences between the pregnancy supplement groups in either sex.

^3^ Females were lighter and shorter than males at birth and throughout infancy (*P*<0.0001). They also gained less weight and length than males by 52 weeks of age (*P*<0.0001). There were no significant differences in SDS between the sexes at any age or change in SDS by 52 weeks.

**Supplemental Table 5.** Mid-upper arm circumference, triceps skinfold thickness and head circumference of female participants at each timepoint by pregnancy supplement group

|  |  | MUAC (cm) | | | | |  | TST (mm) | | | | |  | HC (cm) | | | | |
| --- | --- | --- | --- | --- | --- | --- | --- | --- | --- | --- | --- | --- | --- | --- | --- | --- | --- | --- |
|  |  | F-Ca | |  | F-P | |  | F-Ca | |  | F-P | |  | F-Ca | |  | F-P | |
|  |  | Mean±SD | *n* |  | Mean±SD | *n* |  | Mean±SD | *n* |  | Mean±SD | *n* |  | Mean±SD | *n* |  | Mean±SD | *n* |
| PSO.Y3 |  | 14.9±1.0 | 120 |  | 15.1±1.1 | 126 |  | 9.2±1.8 | 121 |  | 9.5±1.8 | 126 |  | 47.4±1.4 | 119 |  | 47.3±1.3 | 125 |
| PSO.Y5 |  | 15.5±1.1 | 118 |  | 15.5±1.0 | 123 |  | 8.1±1.5 | 117 |  | 8.0±1.3 | 121 |  | 48.8±1.3 | 118 |  | 48.8±1.3 | 123 |
| PSO.Y7 |  | 16.1±1.2 | 119 |  | 16.1±1.3 | 114 |  | 7.5±1.4 | 118 |  | 7.7±1.5 | 116 |  | 50.0±1.3 | 117 |  | 49.8±1.5 | 113 |
| PSE.B |  | 16.4±1.4 | 98 |  | 16.5±1.5 | 97 |  | 7.0±1.7 | 98 |  | 7.2±1.4 | 97 |  | - | - |  | - | - |
| PSO.Y9 |  | 17.1±1.3 | 116 |  | 17.0±1.4 | 115 |  | 6.9±1.6 | 114 |  | 6.9±1.6 | 112 |  | 51.1±1.4 | 115 |  | 50.9±1.5 | 116 |
| PSC.B |  | 17.0±1.4 | 113 |  | 17.3±1.7 | 116 |  | 7.5±1.8 | 113 |  | 7.7±2.2 | 116 |  | - | - |  | - | - |
| PSO.Y11 |  | 19.0±1.5 | 108 |  | 19.3±2.1 | 112 |  | 7.9±2.1 | 109 |  | 8.1±3.0 | 113 |  | 52.0±1.6 | 107 |  | 51.9±1.5 | 109 |
| PSO.Y13 |  | 20.5±1.8 | 39 |  | 21.2±2.2 | 36 |  | 8.7±2.7 | 39 |  | 9.1±2.9 | 36 |  | 52.5±1.6 | 39 |  | 52.9±1.6 | 34 |
| PSC.F1 |  | 21.2±2.4^1^ | 103 |  | 22.2±3.0 | 107 |  | 9.4±3.7^2^ | 102 |  | 10.5±4.1 | 104 |  | - | - |  | - | - |
| PSO.Y15 |  | 23.4±2.6 | 83 |  | 23.9±2.6 | 87 |  | 10.2±3.5 | 81 |  | 11.7±3.7 | 86 |  | 53.8±1.8 | 82 |  | 53.7±1.8 | 85 |
| PSC.F2 |  | 24.0±2.5^2^ | 98 |  | 24.7±3.2 | 99 |  | 13.1±4.8 | 98 |  | 14.3±5.2 | 99 |  | - | - |  | - | - |
| PSO.Y17 |  | 25.0±2.4 | 43 |  | 25.9±3.0 | 46 |  | 12.2±4.4 | 43 |  | 14.1±5.1 | 46 |  | 53.7±1.8 | 43 |  | 54.2±1.8 | 46 |
| PSC.F3 |  | 25.3±2.5 | 94 |  | 25.8±2.9 | 94 |  | 16.0±5.0 | 94 |  | 16.5±6.2 | 94 |  | - | - |  | - | - |

*Abbreviations:* MUAC, mid-upper arm circumference; TST, triceps skinfold thickness; HC, head circumference; F-Ca, females born to mothers in the pregnancy calcium supplement group; F-P, females born to mothers in the pregnancy placebo group; PSO.Y3 to PSO.Y17, Pregnancy Study Offspring Study at timepoints Y3 to Y17; PSE.B, Pregnancy Study Early Nutrition Project; cohort B; PSC.B to PSC.F3, Pregnancy Study Children’s Bone Health, Baseline and Follow-up Studies.

Significance of difference between calcium and placebo groups in cross sectional analysis with age adjustment: **^1^***P* = 0.005, **^2^***P* <0.05

**Supplemental Table 6.** Mid-upper arm circumference, triceps skinfold thickness and head circumference of male participants at each timepoint by pregnancy supplement group

|  |  | MUAC (cm) | | | | |  | TST (mm) | | | | |  | HC(cm) | | | | |
| --- | --- | --- | --- | --- | --- | --- | --- | --- | --- | --- | --- | --- | --- | --- | --- | --- | --- | --- |
|  |  | M-Ca | |  | M-P | |  | M-Ca | |  | M-P | |  | M-Ca | |  | M-P | |
|  |  | Mean±SD | *n* |  | Mean±SD | *n* |  | Mean±SD | *n* |  | Mean±SD | *n* |  | Mean±SD | *n* |  | Mean±SD | *n* |
| PSO.Y3 |  | 15.2±1.2 | 115 |  | 15.2±1.0 | 120 |  | 9.1±1.8**^1^** | 115 |  | 8.6±1.7 | 120 |  | 48.2±1.4 | 115 |  | 48.2±1.4 | 119 |
| PSO.Y5 |  | 15.3±1.1 | 113 |  | 15.2±1.0 | 117 |  | 8.3±1.5 | 114 |  | 8.3±1.7 | 115 |  | 49.6±1.4 | 113 |  | 49.6±1.3 | 116 |
| PSO.Y7 |  | 15.8±1.1 | 109 |  | 15.7±1.1 | 116 |  | 6.9±1.4 | 110 |  | 6.7±1.2 | 116 |  | 50.6±1.4 | 108 |  | 50.4±1.3 | 112 |
| PSE.B |  | 16.1±1.3 | 94 |  | 15.9±1.1 | 98 |  | 5.9±1.4 | 94 |  | 5.7±1.2 | 98 |  | - | - |  | - | - |
| PSO.Y9 |  | 17.2±1.5 | 108 |  | 17.4±1.1 | 109 |  | 6.8±1.6 | 106 |  | 6.9±1.5 | 109 |  | 51.4±1.6 | 109 |  | 51.4±1.3 | 113 |
| PSC.B |  | 16.8±1.4 | 109 |  | 16.6±1.2 | 106 |  | 6.2±1.4 | 109 |  | 5.9±1.2 | 106 |  | - | - |  | - | - |
| PSO.Y11 |  | 18.1±1.5 | 101 |  | 18.1±1.3 | 102 |  | 6.2±1.5 | 101 |  | 6.3±1.6 | 104 |  | 52.1±1.5 | 100 |  | 52.3±1.4 | 103 |
| PSO.Y13 |  | 19.3±1.7 | 31 |  | 19.4±1.0 | 28 |  | 6.5±1.9 | 31 |  | 6.7±1.3 | 28 |  | 52.7±1.6 | 31 |  | 52.5±1.3 | 27 |
| PSC.F1 |  | 19.9±2.3 | 89 |  | 19.7±2.0 | 98 |  | 6.3±1.5 | 89 |  | 6.4±1.6 | 98 |  | - | - |  | - |  |
| PSO.Y15 |  | 21.2±2.0 | 75 |  | 21.2±2.0 | 79 |  | 6.8±1.9 | 75 |  | 6.8±1.8 | 78 |  | 53.1±1.7 | 74 |  | 53.1±1.6 | 79 |
| PSC.F2 |  | 22.7±2.7 | 89 |  | 22.3±2.6 | 92 |  | 6.4±2.6 | 88 |  | 6.3±1.9 | 92 |  | - | - |  | - | - |
| PSO.Y17 |  | 24.0±2.6 | 39 |  | 23.0±2.6 | 40 |  | 7.2±2.7 | 38 |  | 7.1±2.0 | 40 |  | 53.7±2.0 | 39 |  | 54.0±1.7 | 40 |
| PSC.F3 |  | 25.4±2.9**^2^** | 80 |  | 24.4±2.6 | 84 |  | 10.9±6.3 | 79 |  | 9.8±5.7 | 84 |  | - | - |  | - | - |

*Abbreviations:* MUAC, mid-upper arm circumference; TST, triceps skinfold thickness; HC, head circumference; M-Ca, males born to mothers in the pregnancy calcium supplement group; M-P, males born to mothers in the pregnancy placebo group; PSO.Y3 to PSO.Y17, Pregnancy Study Offspring Study at timepoints Y3 to Y17; PSE.B, Pregnancy Study Early Nutrition Project; cohort B; PSC.B to PSC.F3, Pregnancy Study Children’s Bone Health, Baseline and Follow-up Studies.

Significance of difference between calcium and placebo groups in cross-sectional analysis with age adjustment: **^1^***P* = 0.05, **^2^***P* = 0.03

**Supplemental Table 7.** Pubertal status of participants in the PSC series by timepoint, sex and pregnancy supplement group^1^

Study *n* Menses^2^ Breast or genital stage^2^ Pubic hair stage^2^

(%Ca group, %P group) (%Ca group, %P group) (%Ca group, %P group)

1 2 3 4 5 1 2 3 4 5

Females

PSC.B^3^ 112, 119 2, 1 81, 81 16, 13 2, 5 1, 0 0, 1 88, 87 10, 11 2, 2 0, 0 0, 0

PSC.F1 102, 104 22, 32 19, 9 27, 35 28, 28 23, 21 3, 7 24,18 35, 33 26, 31 12, 12 3, 6

PSC.F2 98, 99 80, 84 0, 1 3, 2 21, 23 59, 48 17, 26 0, 3 15, 10 40, 31 41, 49 4, 7

PSC.F3 93, 93 98, 96 0, 0 2, 1 15, 12 47, 43 36, 44 0, 0 3, 2 26, 24 46, 40 25, 34

Males

PSC.B^3^ 109, 107 - 4, 4 37, 36 56, 55 2, 3 1, 2 86, 91 4, 5 0, 0 6, 2 4, 2

PSC.F1 88, 93 - 6, 13 27, 26 49, 42 12, 13 6, 6 52, 56 30, 33 11, 6 2, 4 5, 1

PSC.F2 89, 92 - 2, 1 18, 23 33, 33 12, 14 35, 29 23, 24 58, 56 12, 17 6, 2 1, 1

PSC.F3 93, 93 - 0, 3 24, 13 26, 37 23, 24 27, 23 13, 7 40, 46 33, 30 13, 15 1, 2

^1^There were no significant differences noted between the pregnancy supplement groups in either sex.

^2^Data are % of participants in the pregnancy supplement groups reporting that menstruation had started or were in each Tanner Stage.

^3^Pubertal status in PSC.B was assessed at a later time to the other measurements, at mean age 10.7±1.2 years.

*Abbreviations:* PSC.B to PSC.F3, Pregnancy Study Children’s Bone Health, Baseline and Follow-up Studies; Ca, offspring of mothers supplemented with calcium in pregnancy; P, offspring of mothers in the placebo group in pregnancy.

**Supplemental Table 8.** Dietary intakes of participants in the PSC series by timepoint, sex and pregnancy supplement group^1^

| Study |  | | *n* | | Energy (MJ/d) | | | | |  | | | | | Protein (g/d) | | | Calcium (mg/d) | | | | | | Phosphorus (mg/d) | | | | | | |  |
| --- | --- | --- | --- | --- | --- | --- | --- | --- | --- | --- | --- | --- | --- | --- | --- | --- | --- | --- | --- | --- | --- | --- | --- | --- | --- | --- | --- | --- | --- | --- | --- |
|  | | Ca, P | | | | Ca | | P | | |  | | Ca | | | P | |  | | Ca | | P | | |  | | Ca | | P | |  |
|  | |  | |  | | | Mean±SD | | Mean±SD | | |  | | Mean±SD | | | Mean±SD | |  | | Mean±SD | | Mean±SD | | |  | | Mean±SD | | Mean±SD |  |
| Females | |  | |  | | |  | |  | | |  | |  | | |  | |  | |  | |  | | |  | |  | |  |  |
| PSC.B | | 114, 114 | | | | 5.40±1.30 | | 5.28±1.33 | | |  | | 38.8±10.3 | | | 37.8±10.3 | |  | | 258±121 | | 252±118 | | |  | | 541±155 | | 524±160 | |  |
| PSC.F1 | | 104, 108 | | | | 6.71±1.56 | | 6.56±1.92 | | |  | | 46.0±11.6 | | | 45.1±13.8 | |  | | 298±129 | | 275±126 | | |  | | 657±177 | | 613±186 | |  |
| PSC.F2 | | 90, 94 | | | | 6.72±1.70 | | 7.19±1.85 | | |  | | 48.0±14.2 | | | 51.8±13.5 | |  | | 294±154 | | 320±125 | | |  | | 656±200 | | 692±187 | |  |
| PSC.F3 | | 79, 75 | | | | 6.97±1.75 | | 7.28±2.32 | | |  | | 49.0±15.4 | | | 52.0±18.2 | |  | | 284±112 | | 290±137 | | |  | | 646±183 | | 659±211 | |  |
| Males | |  | |  | | |  | |  | | |  | |  | | |  | |  | |  | |  | | |  | |  | |  |  |
| PSC.B | | 106,105 | | | | 5.92±1.54 | | 5.91±1.54 | | |  | | 42.0±11.5 | | | 42.0±11.5 | |  | | 285±145 | | 285±129 | | |  | | 602±197 | | 592±188 | |  |
| PSC.F1 | | 86, 96 | | | | 7.36±1.58 | | 6.99±1.67 | | |  | | 51.1±13.3 | | | 50.3±14.6 | |  | | 308±160 | | 331±284 | | |  | | 710±198 | | 714±261 | |  |
| PSC.F2 | | 86, 88 | | | | 7.85±2.08 | | 7.65±2.06 | | |  | | 57.0±15.9 | | | 54.8±17.0 | |  | | 363±217 | | 311±170 | | |  | | 793±258 | | 735±224 | |  |
| PSC.F3 | | 67, 63 | | | | 8.30±1.96 | | 8.43±2.13 | | |  | | 59.7±17.1 | | | 58.8±16.9 | |  | | 322±149 | | 352±166 | | |  | | 780±229 | | 778±211 | |  |

*Abbreviations:* PSC.B to PSC.F3, Pregnancy Study Children’s Bone Health, Baseline and Follow-up Studies; Ca, offspring of mothers supplemented with calcium in pregnancy; P, offspring of mothers in the placebo group in pregnancy.

^1^There were no significant differences noted in dietary intakes between the pregnancy supplement groups in either sex.

**Supplemental Table 9.** Differences between female and male participants in blood pressure, height, weight and BMI by timepoint

|  | | Age years | | | | SBP mmHg | | | | |  | DBP mmHg | | | |  | | | Height cm | | | | | |  | | | Weight kg | | | | | | |  | | | | BMI kg/m^2^ | | | | | |
| --- | --- | --- | --- | --- | --- | --- | --- | --- | --- | --- | --- | --- | --- | --- | --- | --- | --- | --- | --- | --- | --- | --- | --- | --- | --- | --- | --- | --- | --- | --- | --- | --- | --- | --- | --- | --- | --- | --- | --- | --- | --- | --- | --- | --- |
|  | |  | | | | | F - M | | | |  | F - M | | | | |  | | | | F - M | | | | |  | | | | F - M | | | | | |  | | | | F - M | | | | |
|  | | Mean±SD | | | ∆±SE | | | | *P* |  | | ∆±SE | | *P* |  | | | ∆±SE | | | | | *P* | |  | | | | ∆±SE | | | *P* | |  | | | | ∆±SE | | | | | *P* |  |
| PSO.Y3 |  | | 3.1±0.1 | -0.2±0.9 | | | | 0.82 | | |  | +0.4±0.8 | 0.60 | | | | | | |  | | -0.8±0.3 | | 0.02 | | |  | | | | -0.5±0.1 | | <0.001 | | | |  | | | | -0.4±0.1 | 0.003 | | |
| PSO.Y5 |  | | 5.0±0.1 | +1.3±0.9 | | | | 0.18 | | |  | +1.9±0.7 | 0.008 | | | | | | |  | | -0.6±0.4 | | 0.14 | | |  | | | | -0.3±0.2 | | 0.04 | | | |  | | | | -0.1±0.1 | 0.45 | | |
| PSO.Y7 |  | | 7.0±0.1 | +2.1±0.9 | | | | 0.02 | | |  | +2.2±0.7 | 0.002 | | | | | | |  | | -0.0±0.4 | | 0.95 | | |  | | | | -0.1±0.2 | | 0.59 | | | |  | | | | -0.1±0.1 | 0.22 | | |
| PSE.B |  | | 7.4±1.2 | +0.8±0.9 | | | | 0.35 | | |  | +1.6±0.8 | 0.03 | | | | | | |  | | +0.3±0.5 | | 0.57 | | |  | | | | -0.3±0.3 | | 0.35 | | | |  | | | | -0.2±0.1 | 0.06 | | |
| PSO.Y9 |  | | 9.0±0.1 | +3.7±0.8 | | | | <0.001 | | |  | +2.4±0.7 | <0.001 | | | | | | |  | | +1.0±0.5 | | 0.02 | | |  | | | | +0.4±0.3 | | 0.14 | | | |  | | | | 0.0±0.1 | 0.88 | | |
| PSC.B |  | | 9.2±0.9 | - | | | | - | | |  | - | - | | | | | | |  | | +1.0±0.5 | | 0.04 | | |  | | | | +0.3±0.3 | | 0.32 | | | |  | | | | -0.1±0.1 | 0.40 | | |
| PSO.Y11 |  | | 11.0±0.2 | +4.1±0.9 | | | | <0.001 | | |  | +1.9±0.8 | 0.01 | | | | | | |  | | +2.1±0.6 | | <0.001 | | |  | | | | +1.3±0.4 | | 0.002 | | | |  | | | | +0.2±0.2 | 0.20 | | |
| PSO.Y13 |  | | 13.0±0.2 | +7.1±1.6 | | | | <0.001 | | |  | +5.1±1.5 | <0.001 | | | | | | |  | | +4.3±1.2 | | <0.001 | | |  | | | | +4.3±1.0 | | <0.001 | | | |  | | | | +0.9±0.3 | 0.004 | | |
| PSC.F1 |  | | 13.8±1.2 | +5.1±1.0 | | | | <0.001 | | |  | +3.9±0.9 | <0.001 | | | | | | |  | | +3.7±0.7 | | <0.001 | | |  | | | | +4.9±0.7 | | <0.001 | | | |  | | | | +1.3±0.2 | <0.001 | | |
| PSO.Y15 |  | | 15.0±0.1 | +4.1±1.1 | | | | <0.001 | | |  | +3.5±0.9 | <0.001 | | | | | | |  | | +2.2±0.8 | | 0.008 | | |  | | | | +6.5±0.9 | | <0.001 | | | |  | | | | +2.2±0.3 | <0.001 | | |
| PSC.F2 |  | | 16.3±1.2 | +1.6±1.0 | | | | 0.11 | | |  | +3.8±0.8 | <0.001 | | | | | | |  | | -3.1±0.7 | | <0.001 | | |  | | | | +3.3±0.8 | | <0.001 | | | |  | | | | +2.1±0.3 | <0.001 | | |
| PSO.Y17 |  | | 17.0±0.9 | +1.4±1.5 | | | | 0.32 | | |  | +3.7±1.2 | 0.002 | | | | | | |  | | -6.3±1.0 | | <0.001 | | |  | | | | +3.1±1.3 | | 0.02 | | | |  | | | | +2.6±0.4 | <0.001 | | |
| PSC.F3 |  | | 18.4±1.4 | -3.6±1.0 | | | | <0.001 | | |  | +2.0±0.8 | 0.02 | | | | | | |  | | -9.5±0.7 | | <0.001 | | |  | | | | -1.6±0.9 | | 0.06 | | | |  | | | | +1.8±0.3 | <0.001 | | |

Measurement data are mean difference (∆) ± SE for difference between female and male participants without splitting by pregnancy supplement group, the *P* value indicates the significance of the difference obtained by analysis of covariance with age adjustment*.* Blood pressure was measured using Dinamap 8100 in the PSO series and Omron 7051T in PSE.B and the PSC series. Blood pressure was not measured at timepoint PSC.B. The total numbers per measurement in each sex with the pregnancy supplement groups combined can be obtained from Tables 1-4 in the main paper.

*Abbreviations:* SBP, systolic blood pressure; DBP, diastolic blood pressure; BMI, body mass index;; F, females born to mothers in the pregnancy calcium supplement trial; M, males born to mothers in the pregnancy calcium supplement trial; PSO.Y3 to PSO.Y17, Pregnancy Study Offspring Study at timepoints Y3 to Y17; PSE.B, Pregnancy Study Early Nutrition Project; cohort B; PSC.B to PSC.F3, Pregnancy Study Children’s Bone Health, Baseline and Follow-up Studies.

**Supplemental Table 10.** Differences between female and male participants in mean mid-upper arm circumference, triceps skinfold thickness and head circumference by timepoint.

|  | |  | |  |  | | | | | | | | | | | |  | | | | | | | | | |  |
| --- | --- | --- | --- | --- | --- | --- | --- | --- | --- | --- | --- | --- | --- | --- | --- | --- | --- | --- | --- | --- | --- | --- | --- | --- | --- | --- | --- |
|  | | Age years | | | | | | MUAC cm | | |  | | | | TST mm | | | | |  | HC cm | | | | |  | |
|  | |  | | | | | | | F - M | | |  | | F - M | | | | |  | | F - M | | | | | |  |
|  | | Mean±SD | | | | | ∆±SE | | | *P* |  | | ∆±SE | | | | | *P* | |  | ∆±SE | | | *P* | | |  |
| PSO.Y3 |  | | 3.1±0.1 | | | -0.2±0.1 | | | | 0.04 | | |  | | | +0.5±0.2 | | 0.001 | | | |  | -0.9±0.1 | | <0.001 | |  |
| PSO.Y5 |  | | 5.0±0.1 | | | +0.2±0.1 | | | | 0.03 | | |  | | | -0.2±0.1 | | 0.14 | | | |  | -0.8±0.1 | | <0.001 | |  |
| PSO.Y7 |  | | 7.0±0.1 | | | +0.4±0.1 | | | | <0.001 | | |  | | | +0.8±0.1 | | <0.001 | | | |  | -0.6±0.1 | | <0.001 | |  |
| PSE.B |  | | 7.4±1.2 | | | +0.4±0.1 | | | | 0.002 | | |  | | | +1.3±0.2 | | <0.001 | | | |  | - | | - | |  |
| PSO.Y9 |  | | 9.0±0.1 | | | -0.2±0.1 | | | | 0.06 | | |  | | | 0.0±0.2 | | 0.9 | | | |  | -0.4±0.1 | | 0.005 | |  |
| PSC.B |  | | 9.2±0.9 | | | +0.5±0.1 | | | | <0.001 | | |  | | | +1.5±0.2 | | <0.001 | | | |  | - | | - | |  |
| PSO.Y11 |  | | 11.0±0.2 | | | +1.0±0.2 | | | | <0.001 | | |  | | | +1.8±0.2 | | <0.001 | | | |  | -0.2±0.2 | | 0.13 | |  |
| PSO.Y13 |  | | 13.0±0.2 | | | +1.5±0.3 | | | | <0.001 | | |  | | | +2.3±0.4 | | <0.001 | | | |  | +0.1±0.3 | | 0.72 | |  |
| PSC.F1 |  | | 13.8±1.2 | | | +1.9±0.3 | | | | <0.001 | | |  | | | +3.6±0.3 | | <0.001 | | | |  | - | | - | |  |
| PSO.Y15 |  | | 15.0±0.1 | | | +2.5±0.3 | | | | <0.001 | | |  | | | +4.2±0.3 | | <0.001 | | | |  | +0.6±0.2 | | 0.002 | |  |
| PSC.F2 |  | | 16.3±1.2 | | | +1.9±0.3 | | | | <0.001 | | |  | | | +7.4±0.4 | | <0.001 | | | |  | - | | - | |  |
| PSO.Y17 |  | | 17.0±0.9 | | | +2.0±0.4 | | | | <0.001 | | |  | | | +6.1±0.6 | | <0.001 | | | |  | +0.1±0.3 | | 0.67 | |  |
| PSC.F3 |  | | 18.4±1.4 | | | +0.6±0.3 | | | | 0.03 | | |  | | | +5.9±0.6 | | <0.001 | | | |  | - | | - | |  |

Measurement data are mean difference (∆) ± SE for difference between female and male participants without splitting by pregnancy supplement group, the *P* value indicates the significance of the difference obtained by analysis of covariance with age adjustment*.* Head circumference was not measured in the PSC series. The total numbers per measurement in each sex with the pregnancy supplement groups combined can be obtained from Supplemental Tables 5 and 6.

*Abbreviations:* MUAC, mid-upper arm circumference; TST, triceps skinfold thickness; HC, head circumference; F, females born to mothers in the pregnancy calcium supplement trial; M, males born to mothers in the pregnancy calcium supplement trial; PSO.Y3 to PSO.Y17, Pregnancy Study Offspring Study at timepoints Y3 to Y17; PSE.B, Pregnancy Study Early Nutrition Project; cohort B; PSC.B to PSC.F3, Pregnancy Study Children’s Bone Health, Baseline and Follow-up Studies.

**Supplemental Table 11.** Height and weight SD-scores of female participants at each timepoint by pregnancy supplement group

|  |  | Height SDS | | | | | | | | | |  | | | Weight SDS | | | | | | | | | | | | |  | | |  |
| --- | --- | --- | --- | --- | --- | --- | --- | --- | --- | --- | --- | --- | --- | --- | --- | --- | --- | --- | --- | --- | --- | --- | --- | --- | --- | --- | --- | --- | --- | --- | --- |
|  |  | F-Ca | |  | | | F-P | | | | |  | | | F-Ca | | | | |  | | | F-P | | | | |  | | |  |
|  |  | Mean±SD | *n* | |  | | | Mean±SD | | *n* | | |  | | | Mean±SD | | *n* | | |  | | | Mean±SD | | *n* | | |  | | |
| PSO.Y3 |  | -1.88±0.97 | 121 | | |  | | | -1.84±1.08 | | 125 | | |  | | | -1.75±1.09 | | 121 | | |  | | | -1.65±1.08 | | 125 | | |  | |
| PSO.Y5 |  | -1.49±0.93 | 116 | | |  | | | -1.41±1.03 | | 121 | | |  | | | -1.69±1.01 | | 117 | | |  | | | -1.60±1.08 | | 122 | | |  | |
| PSO.Y7 |  | -1.19±0.83 | 112 | | |  | | | -1.09±1.02 | | 111 | | |  | | | -1.48±0.92 | | 119 | | |  | | | -1.52±1.04 | | 114 | | |  | |
| PSE.B |  | -1.05±0.79 | 99 | | |  | | | -0.89±1.01 | | 97 | | |  | | | -1.65±0.92 | | 99 | | |  | | | -1.50±1.00 | | 97 | | |  | |
| PSO.Y9 |  | -1.06±0.79 | 112 | | |  | | | -0.98±0.93 | | 114 | | |  | | | -1.48±0.84 | | 116 | | |  | | | -1.36±1.00 | | 116 | | |  | |
| PSC.B |  | -1.09±0.81^1^ | 114 | | |  | | | -0.86±0.86 | | 115 | | |  | | | -1.54±0.80 | | 114 | | |  | | | -1.34±0.91 | | 117 | | |  | |
| PSO.Y11 |  | -0.98±0.79 | 109 | | |  | | | -0.85±0.98 | | 112 | | |  | | | -1.26±0.79 | | 109 | | |  | | | -1.17±1.00 | | 113 | | |  | |
| PSO.Y13 |  | -0.92±1.07 | 39 | | |  | | | -0.49±1.09 | | 36 | | |  | | | -1.49±1.14 | | 40 | | |  | | | -1.19±1.18 | | 36 | | |  | |
| PSC.F1 |  | -0.89±0.86^2^ | 104 | | |  | | | -0.58±1.01 | | 107 | | |  | | | -1.41±1.04^2^ | | 104 | | |  | | | -1.03±1.20 | | 107 | | |  | |
| PSO.Y15 |  | -0.88±0.97 | 83 | | |  | | | -0.74±1.01 | | 85 | | |  | | | -1.18±1.28 | | 83 | | |  | | | -0.92±1.29 | | 87 | | |  | |
| PSC.F2 |  | -0.65±0.93 | 98 | | |  | | | -0.45±0.94 | | 99 | | |  | | | -1.05±1.13 | | 98 | | |  | | | -0.81±1.28 | | 99 | | |  | |
| PSO.Y17 |  | -0.73±0.93 | 42 | | |  | | | -0.67±0.96 | | 46 | | |  | | | -0.94±1.02 | | 43 | | |  | | | -0.51±1.21 | | 46 | | |  | |
| PSC.F3 |  | -0.50±0.96 | 94 | | |  | | | -0.41±0.89 | | 94 | | |  | | | -0.71±1.04 | | 94 | | |  | | | -0.60±1.12 | | 94 | | |  | |

Data are mean ± SD SD-score using UK reference data for females (Freeman JV *et al*, Archives of Diseases in Childhood 1995;73:17-24). Significance of difference between pregnancy supplement groups by analysis of variance ^1^*P* = 0.04, ^2^*P* = 0.01

*Abbreviations*: SDS, SD-score; F-Ca, females born to mothers in the pregnancy calcium supplement group; F-P, females born to mothers in the pregnancy placebo group; PSO.Y3 to PSO.Y17, Pregnancy Study Offspring Study at timepoints Y3 to Y17; PSE.B, Pregnancy Study Early Nutrition Project; cohort B; PSC.B to PSC.F3, Pregnancy Study Children’s Bone Health, Baseline and Follow-up Studies.

**Supplemental Table 12.** Height and weight SD scores of male participants at each timepoint by pregnancy supplement group^1^

|  |  | Height SDS | | | | |  | Weight SDS | | | | |  |
| --- | --- | --- | --- | --- | --- | --- | --- | --- | --- | --- | --- | --- | --- |
|  |  | M-Ca | |  | M-P | |  | M-Ca | |  | M-P | |  |
|  |  | Mean±SD | *n* |  | Mean±SD | *n* |  | Mean±SD | *n* |  | Mean±SD | *n* |  |
| PSO.Y3 |  | -1.86±0.99 | 114 |  | -1.89±0.99 | 118 |  | -1.67±1.16 | 113 |  | -1.59±0.96 | 120 |  |
| PSO.Y5 |  | -1.48±1.01 | 111 |  | -1.43±0.90 | 112 |  | -1.74±1.14 | 114 |  | -1.76±0.99 | 117 |  |
| PSO.Y7 |  | -1.30±0.88 | 105 |  | -1.24±0.89 | 111 |  | -1.69±1.03 | 110 |  | -1.71±0.91 | 116 |  |
| PSE.B |  | -1.18±0.84 | 94 |  | -1.11±0.85 | 98 |  | -1.62±1.00 | 94 |  | -1.72±0.93 | 99 |  |
| PSO.Y9 |  | -1.25±0.85 | 108 |  | -1.32±0.80 | 114 |  | -1.53±0.98 | 111 |  | -1.69±0.99 | 114 |  |
| PSC.B |  | -1.15±0.88 | 109 |  | -1.22±0.80 | 107 |  | -1.52±1.03 | 109 |  | -1.63±0.91 | 107 |  |
| PSO.Y11 |  | -1.12±0.92 | 100 |  | -1.16±0.85 | 103 |  | -1.38±0.97 | 101 |  | -1.43±0.86 | 104 |  |
| PSO.Y13 |  | -1.13±0.89 | 31 |  | -1.16±0.77 | 28 |  | -1.79±0.92 | 31 |  | -1.66±0.62 | 28 |  |
| PSC.F1 |  | -1.37±0.98 | 89 |  | -1.47±0.92 | 98 |  | -1.76±1.06 | 89 |  | -1.91±0.93 | 98 |  |
| PSO.Y15 |  | -1.74±1.04 | 75 |  | -1.72±1.02 | 77 |  | -1.98±1.08 | 75 |  | -1.97±1.03 | 79 |  |
| PSC.F2 |  | -1.30±1.09 | 89 |  | -1.39±1.09 | 92 |  | -1.82±1.23 | 89 |  | -2.03±1.12 | 92 |  |
| PSO.Y17 |  | -1.40±0.98 | 38 |  | -1.49±1.02 | 39 |  | -1.94±1.45 | 39 |  | -2.32±1.51 | 40 |  |
| PSC.F3 |  | -0.93±1.02 | 80 |  | -0.90±1.13 | 84 |  | -1.47±1.28 | 80 |  | -1.82±1.32 | 84 |  |

Data are SD-scores using UK reference data for males (Freeman JV *et al*, Archives of Diseases in Childhood 1995;73:17-24).

^1^There were no significant differences noted in SD-scores between the pregnancy supplement groups.

*Abbreviations*: SDS, SD-score; M-Ca, males born to mothers in the pregnancy calcium supplement group; M-P, males born to mothers in the pregnancy placebo group; PSO.Y3 to PSO.Y17, Pregnancy Study Offspring Study at timepoints Y3 to Y17; PSE.B, Pregnancy Study Early Nutrition Project; cohort B; PSC.B to PSC.F3, Pregnancy Study Children’s Bone Health, Baseline and Follow-up Studies.
